# Deep learning-based predictions and analysis of Norovirus dynamics using a fractional-order mathematical model

**DOI:** 10.1371/journal.pone.0359983

**Published:** 2026-10-14

**Authors:** Basit Rehman, Akhtar Ali

**Affiliations:** Department of Mathematics, Government College University, Faisalabad, Pakistan; University of Lakki Marwat, PAKISTAN

## Abstract

Norovirus, which is extremely contagious and has a significant impact on the gastrointestinal tract, has also been one of the main potential threats to health in the population. The use of fractional calculus has received much interest in the past few years with regard to the modeling of infectious diseases, especially because it allows modeling the memory and nonlocal processes in a dynamic system. In this study, a mathematical model is developed to investigate the transmission dynamics of norovirus. The proposed model consists of five epidemiological compartments: susceptible, exposed, infected, vaccinated, and recovered populations. The existence and uniqueness of the mathematical model are proven by a fixed-point theorem. The findings demonstrate that the sustainability of the disease-free equilibrium increases with a decrease in fractional order. In order to observe the effects of the fractional order on the dynamics of disease, numerical simulations are carried out based on the three-step Adams-Bashforth procedure, which supports the theoretical results with graphical illustrations. Moreover, the results are validated through the comparison with deep neural networks. The comprehensive nature of numerical simulations carried out at different fractional orders and transmission parameters indicates that the dynamics of fractional orders are effective in describing memory and heterogeneity of the disease transmission, which are characterized by classical integer-order models only to a limited extent. The results also indicate that the high coverage of vaccination and improvement in the effectiveness of treatment will greatly decrease the prevalence of infections. Further, the model has been demonstrated to be numerically stable and positive in real parameter disclosures. Altogether, this study provides a reliable mathematical approach and better understanding for analyzing disease dynamics and may be extended to other applications in epidemiology and related scientific fields.

## 1. Introduction

Norovirus outbreaks represent a significant issue in the health care of the population and are associated with acute gastroenteritis in persons of all ages. Millions of cases are documented every year around the world, with a significant contribution to global morbidity and imposing a substantial load on the health care system. Norovirus is transmitted by several different methods, as the souring of eatables and water and contact with infected surfaces and persons. Vomitus also contributes greatly to the transmission via aerosolized particles. The probability of food contamination is impacted by a number of factors, such as food hygiene behaviours of food handlers, environmental viral survival, shedding time, and inactivation rate of the virus [1]. Most often, outbreaks can occur in semi-closed facilities or surroundings like schools [2,3], nursing homes, hospitals, restaurants, and cruise ships, where close contact makes it easier to pass the infection. The disease usually heals in two to three days among healthy people; however, when it is severe, it can cause severe dehydration among children, elderly people, and patients with compromised immunity, which can require medical attention [4]. Norovirus is considered to be among the major causes of acute gastroenteritis and has a close relationship with morbidity, mortality, and healthcare spending. The reported cases show a significant seasonality, with more than half of the reported cases in the colder months. This trend can be associated with ecological and demographically; to name a few, norovirus can survive better in colder temperatures and can be transmitted more easily after an increase in rainfall [5], and thus this trend repeats itself after such an increase in precipitation occurs [6–8].

Moreover, various vaccines have already been investigated, with one of them being developed by Takeda Pharmaceuticals, being on the proof-of-concept stage [9]. Considerable efforts are currently underway to develop an effective vaccine against norovirus. Owing to the high genetic diversity of the virus and the absence of long-lasting natural immunity, vaccine development remains challenging. Nevertheless, several vaccine candidates are undergoing clinical evaluation and have shown encouraging results, offering hope for reducing the global burden of norovirus infections, particularly among children and adolescents [10,11].

Researchers are also turning to mathematical modeling and big medical datasets to estimate norovirus prevalence and guide intervention measures to overcome cases of direct surveillance. Mathematical models offer a scientific manner of comprehending the dynamics of infectious diseases, whether in small-scale outbreaks, such as in hospitals, or the epidemics that affect the entire community [12]. One of the most important metrics based on these models is the basic reproduction number, which reflects the possible spread of an epidemic and informs the common control of the population [13,14]. Mathematical modeling is the key to the study on the transformation of norovirus, especially due to its rapid spread, low infectious dose, and frequent outbreaks in confined spaces. A fractional-order model using the Caputo-Fabrizio operator is used to capture the effects of memory and nonlocal transmission dynamics, and it represents a more realistic model of the processes than integer-order models. The findings can be used by health authorities to estimate strategies, including the increase in vaccination coverage and treatment advancements, which would eventually help in the effective prevention and control of outbreaks. These models take into consideration various parameters like the dynamics of transmission, population structure, and environmental factors, which enable them to predict the pattern of the outbreak and also assess the mitigation measures. They also help to estimate the contribution of risk by different exposure pathways, such as contaminated surfaces and aerosols, in addition to predictions of risks [15,16]. Symmetry-based epidemic models can be used to understand the impact of demographic and spatial distributions on susceptible, infected, and recovered populations. Since there are spatial and temporal symmetries that affect disease spread, they can be explicitly addressed in the model design directly [17],.

Fractional calculus has been receiving more and more interest because it is possible to model systems with memory and sophisticated internal behaviour using this technique, as compared to integer-order models [18,19]. The methods, including the Adams-Bashforth, predictor-corrector, and the series solutions, approximately solve non-integer-order systems. The extensions of ordinary derivatives to non-integer orders are called the fractional derivatives, and they are characterized by operators like the Riemann-Liouville integral [20]. The tools have been used in various applications, such as blood flow modeling, diffusion in porous media, and the spreading of infectious diseases [21]. Moreover, a fractional-order mathematical model was proposed to study the transmission dynamics of norovirus. The model was analyzed for its qualitative properties, including existence, uniqueness, positivity, boundedness, and stability, and was solved using a GL-Non-Standard Finite Difference (GL-NSFD) scheme. They highlighted the significance of fractional-order modeling in providing a more realistic description of norovirus epidemic dynamics and supporting public health decision-making [22]. A fractional-order mathematical model of norovirus transmission that incorporates both vaccination and asymptomatic carriers was investigated by [23]. The model is formulated using a fractional derivative to account for memory effects in the transmission process. A compartmental mathematical model was developed to study norovirus transmission dynamics by incorporating environmental transmission. The stability analysis of disease-free and endemic equilibria was established using Lyapunov methods. Sensitivity and cost-effectiveness analyses revealed that environmental sanitation is a key and economically efficient strategy for reducing outbreaks [24].

Furthermore, artificial neural networks (ANNs), and more recently deep neural networks (DNNs), have become powerful tools for approximating solutions of differential equations arising in mathematical epidemiology [25]. Many researchers have successfully applied DNN-based methods to a wide range of nonlinear dynamical systems, including epidemiological models, demonstrating high accuracy and flexibility in capturing complex temporal behaviors [26,27]. A feedforward neural network approach was developed which provide an efficient framework for solving nonlinear systems of ordinary differential equations without relying on traditional discretization schemes [28]. An ANN-based deep collocation method was developed and compared to classical numerical methods which shows that DNN-based approaches yield smooth and differentiable solutions over the entire domain, often represented in closed functional forms [29].

Motivated by these developments [19,22,23], the present work employs a deep neural network to approximate the solution of the norovirus SVEIR model. The proposed approach leverages the strengths of DNNs in function approximation, combined with the ability to enforce the underlying system dynamics through the loss function, thereby providing an accurate and efficient alternative when compared with traditional numerical methods. The paper has been structured as follows. In the first Section, the development of the proposed fractional-order norovirus model has been presented. Section 3 offers an analytical framework and mathematical preliminaries that will be used in the study. In Section 4, the key theoretical findings are given, which prove the uniqueness and existence of the solutions in the context of the fixed-point theory and explore the stability properties of the solutions therein. Section 5 presents the procedure of the three-step Adams-Bashforth scheme together with numerical simulations performed on the selected method, where MATLAB was implemented and graphically illustrated. Section 6 continues the analysis including numerical simulations and discussions using a deep neural network approach that helps to bring additional insights into the dynamics of the model. Lastly, Section 7 ends the work with a summary of the main findings and recommendations for future research.

## 2. Model formulation

The transmission dynamics of norovirus are described using a compartmental framework that categorizes the population into susceptible (*S*), vaccinated (*V*), exposed (*E*), infected (*I*), and recovered (*R*) groups. Individuals enter the susceptible class at a constant recruitment rate Λ. Susceptible individuals may become infected through effective contact with infectious individuals at a transmission rate β. Vaccination occurs at a rate θ, transferring individuals into the vaccinated class, where they acquire partial protection characterized by vaccine efficacy ϵ. However, this protection may wane over time, and vaccinated individuals can return to the susceptible class at a rate τ.

Following exposure, individuals move into the latent (exposed) class (*E*) and progress to the infectious class (*I*) at a rate δ. Infected individuals recover at a rate ω, gaining immunity and entering the recovered class (*R*). Additionally, infected individuals may experience disease-induced mortality at rate *d*, while all compartments are subject to natural mortality at rate μ. Recovered individuals are assumed to possess immunity against reinfection.

Overall, the model captures the interactions among different population groups and emphasizes the roles of transmission, vaccination, immunity loss, and recovery in shaping the spread of Norovirus. The complete system dynamics are governed by the set of differential equations presented in Equation 1.


$$\begin{array}{cc} \frac{dS}{dt} & =\Lambda +\tau V-(\beta I+\theta +\mu )S,\\ \frac{dV}{dt} & =\theta S-\left((1-\epsilon )\beta I+\tau +\mu \right)V,\\ \frac{dE}{dt} & =\beta IS+(1-\epsilon )\beta IV-(\delta +\mu )E,\\ \frac{dI}{dt} & =\delta E-(\omega +d+\mu )I,\\ \frac{dR}{dt} & =\omega I-\mu R. \end{array}$$ (1)

with initial conditions:


$$\begin{array}{c} S(0)>0,\;E(0)\geq 0,\;V(0)\geq 0,\;I(0)\geq 0,\;R(0)\geq 0. \end{array}$$

Moreover, the first-order time derivatives in the classical SEIVR model in equation 1 are replaced by the Caputo–Fabrizio fractional derivative ${\;}^{CF}D_{t}^{\zeta }$, where 0<ζ≤1. Consequently, the resulting fractional-order model provides a more comprehensive framework for analyzing the spread of the disease and evaluating the effects of vaccination, recovery, and other epidemiological parameters under memory-dependent dynamics. The schematic diagram for the proposed model is represented in Fig 1.

**Fig 1:**
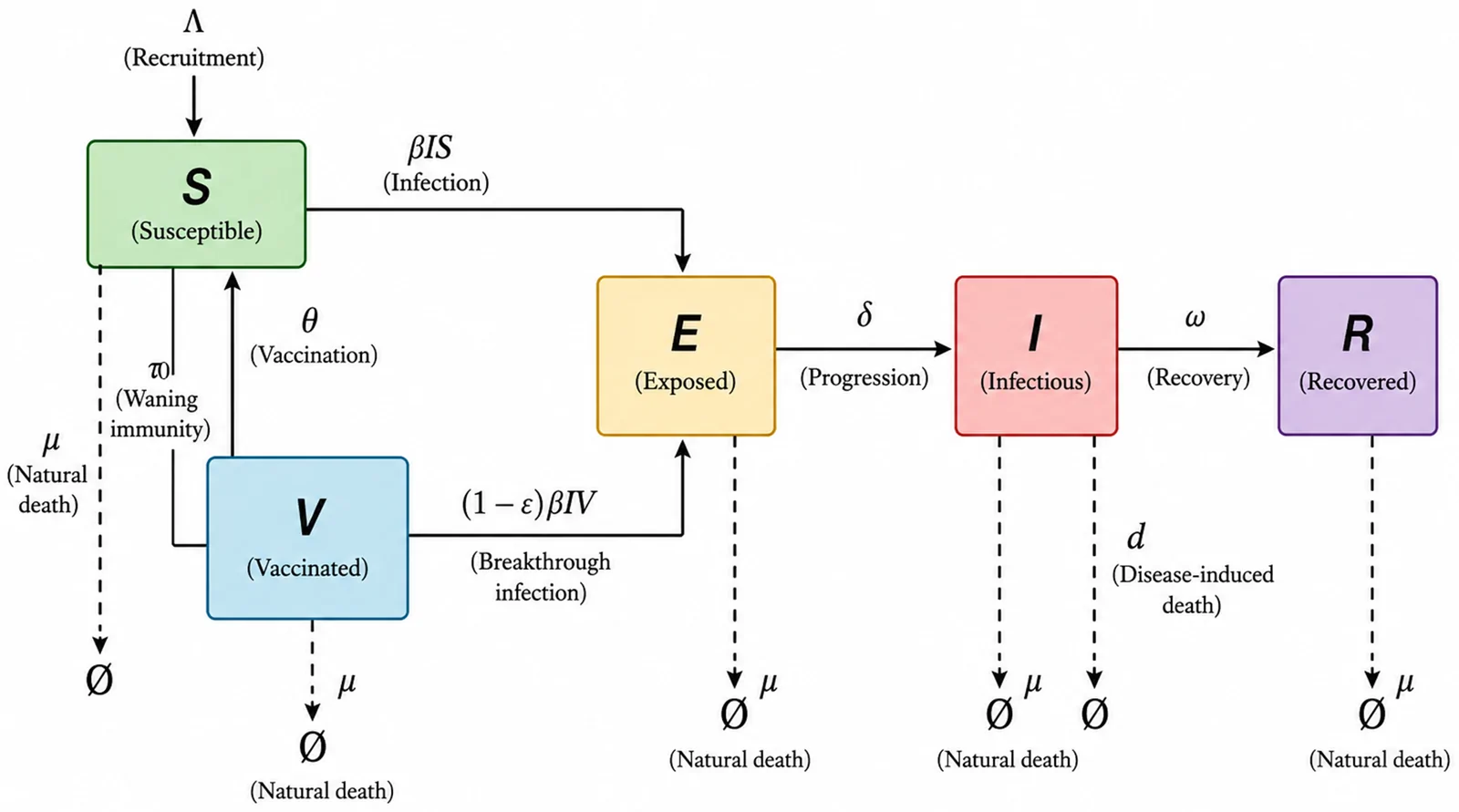
Schematic diagram of the model.

The model parameters used in this study, along with their description are listed in Table 1 [23].

**Table 1:** Provides a detailed description of the parameters and their respective values employed in the proposed model.

Parameter	Description	Value	Source
ω	Treatment rate	0.8378/per day	Assumed
β	Effective contact rate	0.0178/per day	Assumed
*d*	Disease-induced death rate	0.02/per day	[23]
Λ	The rate of Susceptible population Recruited	0.4636/per day	Assumed
τ	Rate of decline of immunity in the vaccinated population	0.1/per day	Assumed
δ	Rate of exposed to infectious	0.0078/per day	Assumed
ϵ	Vaccine efficacy	0.94/per day	Estimated
μ	Rate of Natural death	0.0004/per day	Assumed
θ	Rate of Vaccination	0.54/per day	Assumed

## 3. Basic definitions

This section contains the essential fractional differential operators along with their attributes. Moreover, for analysing the stability, uniqueness, and existence of the solution, a few fixed point theorems and spaces will be used.

**Definition 2.1.** If $\tilde{X}$ is a Banach space, then $F\subset C(\tilde{X})$ is equicontinuous if $\forall \epsilon >0,\;\;\;\;\forall \tilde{a},\tilde{b}\in \tilde{X}$:


$$\begin{array}{c} \|\tilde{a}-\tilde{b}\|<\delta \;\Rightarrow \;\|𝒦(\tilde{a})-𝒦(\tilde{b})\|<\epsilon ,\;\;\;\exists \delta >0\;\;\;\forall 𝒦\in F \end{array}$$

[30]

**Definition 2.2.** Consider a bounded subset *F* of *A.* A map 𝒦:A→A is said to be completely continuous if the set 𝒦(F) is relatively compact [30].

**Theorem 2.1.** *(Arzela-Ascoli theorem) Let* $\tilde{X}$ *be a compact space. If F is an equicontinuous and bounded subset of* $C(\tilde{X})$*, then the operator F is relatively compact* [30]*.*

**Theorem 2.2.** *(Schauder’s fixed point theorem* [30]) *Consider a complete metric space* $\left(\tilde{X},d\right)$ *with a closed convex subset D*_*1*_ *of* $\tilde{X}$*. Let* $𝒦:D_{1}\to D_{1}$ *is relatively compact; then there is at least one fixed point* $x^{*}\in D_{1}$ *for the operator* 𝒦 *such that*


$$\begin{array}{c} 𝒦x^{*}=x^{*}. \end{array}$$ (2)

**Theorem 2.3.** *(Krasnoselskii’s fixed point theorem* [31]) *Assume that D*_*1*_ *is a nonempty closed convex subset of a Banach space* $\tilde{X}$*,L*_*1*_ *and L*_*2*_ *are two operators that map D*_*1*_ *into* $\tilde{X}$*. Such that:*

1. $L_{1}v_{1}+L_{2}z\in D_{1}$ $\forall z\in D_{1}$,
2. *L*_*1*_ *is a contraction, and*
3. *L*_*2*_ *is compact and continuous.*

*Then,* $L_{1}v_{1}+L_{2}v_{1}=v_{1}$ *has at least one solution.*

**Definition 2.3.** If $f_{1}\in H^{-1}(a,b)=f_{1}:f_{1}\in L^{2}(a,b)$ and $f_{1}\in L^{2}(a,b)$ and ζ∈[0,1] ([32]), then the Caputo-Fabrizio fractional derivative is defined as:


$$\begin{array}{c} {\;}^{CF}D_{t}^{\zeta }\left(f_{1}(t)\right)=\frac{M(\zeta )}{1-\zeta }\int_{a}^{t}f_{1}^{{\;}^{\prime }}(𝒦)exp[-\zeta \frac{t-𝒦}{1-\zeta }]d𝒦. \end{array}$$ (3)

Here M(ζ) is labeled as a normalization function with M(0)=M(1)=1 a normalization property.

By Definition 2.3, if ${\;}^{CF}D^{\zeta }$ is zero, *f*_1_(*t*) is constant, but *t*his is not a guarantee that the kernel has a singularity for *t* = *T*.

**Definition 2.4.** ([32]) If ζ∈[0,1] and any $f_{1}\in L^{-1}(-\infty ,b)$, equation (3) can be defined as:


$$\begin{array}{c} {\;}^{CF}D_{-\infty }^{\zeta }\left(f_{1}(t)\right)=\frac{\zeta M(\zeta )}{1-\zeta }\int_{-\infty }^{t}(f_{1}(t)-f_{1}(𝒦))exp[-\zeta \frac{t-𝒦}{1-\zeta }]d𝒦, \end{array}$$

Losada and Nieto worked on the improvement of the Caputo-Fabrizio fractional derivative, which is to be defined as:


$$\begin{array}{c} {\;}^{CF}D_{t}^{\zeta }\left(f_{1}(t)\right)=\frac{\left(2-\zeta \right)M(\zeta )}{2(1-\zeta )}\int_{a}^{t}f_{1}^{{\;}^{\prime }}(𝒦)exp[-\zeta \frac{t-𝒦}{1-\zeta }]d𝒦. \end{array}$$ (4)

Later, equation 3 was used to define the fractional integral in equation 5 as follows.

**Definition 2.5.** Consider 0<ζ<1. The fractional Caputo-Fabrizio integral of a function *f*_*1*_ order ζ is


$$\begin{array}{c} {\;}^{CF}I_{t}^{\zeta }\left(f_{1}(t)\right)=\frac{2(1-\zeta )}{M(\zeta )(2-\zeta )}f_{1}(t)+\frac{2\zeta }{M(\zeta )(2-\zeta )}\int_{0}^{t}f_{1}(𝒦)d𝒦,\;\;t\geq 0. \end{array}$$ (5)

**Remark 2.4.** *Consider the Caputo-Fabrizio type fractional integral for function f*_*1*_ *with order* 0<ζ<1*. It will be a mean between its first-order integral and the function f*_*1*_*, then by Eq. 5*,


$$\begin{array}{c} \frac{2(1-\zeta )}{M(\zeta )(2-\zeta )}+\frac{2\zeta }{M(\zeta )(2-\zeta )}=1, \end{array}$$ (6)

*and therefore* $M(\zeta )=\frac{2}{2-\zeta },\;\;\;0<\zeta <1$.

*A new Caputo-Fabrizio derivative and its associated integral by using* $M(\zeta )=\frac{2}{2-\zeta }$ *M (ζ) = 2 2 − ζ are proposed by Losada and Nieto.*

**Definition 2.6.** [32] Consider 0<ζ<1. The fractional Caputo-Fabrizio derivative of a function *f*_*1*_ of order ζ is


$$\begin{array}{c} {\;}^{CF}D_{t}^{\zeta }\left(f_{1}(t)\right)=\frac{1}{1-\zeta }\int_{a}^{t}f_{1}^{{\;}^{\prime }}(𝒦)exp[-\zeta \frac{t-𝒦}{1-\zeta }]d𝒦,\;\;t\geq 0, \end{array}$$ (7)

and its related fractional integral may be written as:


$$\begin{array}{c} {\;}^{CF}I_{t}^{\zeta }\left(f_{1}{)}_{(}t)\right)=\left(1-\zeta \right)f_{1}(t)+\zeta \int_{0}^{t}f_{1}(𝒦)d𝒦,\;\;t\geq 0, \end{array}$$ (8)

Such that


$$\begin{array}{c} ({\;}^{CF}I_{0}^{\zeta })({\;}^{CF}D_{t}^{\zeta }\left(f_{1}(t)\right))=f_{1}(t)-f_{1}(0). \end{array}$$ (9)

The main difference between the old Caputo operator and with the Caputo-Fabrizio operator is related to the new kernel that there is no singularity for *t* = *T*.

**Lemma 2.5.** [33] *The solution of the initial value problem is given as:*


$$\begin{array}{cc} {\;}^{CF}D_{t}^{\zeta }\left(v(t)\right)= & \psi (t)\;\;t\geq 0,\;\;\;0<\zeta <1,\\ v(0)= & v_{0}, \end{array}$$ (10)

$$\begin{array}{c} v(t)=v_{0}+\frac{2(1-\zeta )}{M(\zeta )(2-\zeta )}(\psi (t)-\psi (0))+\frac{2\zeta }{M(\zeta )(2-\zeta )}\int_{0}^{t}\psi (𝒦)d𝒦. \end{array}$$ (11)

## 4. Main results

This section explains the uniqueness and existence of the proposed model and the stability of its solution. Let us define a notation that is used in a subsequent theorem.


$$\begin{array}{c} \tilde{X}=\{v\in C([0,T],R^{5}):\|v\|<\infty \}, \end{array}$$

The Banach space with the norm


$$\begin{array}{c} \|v\|=\|S,V,E,I,R\|=max_{t\in [0,T]}\{|S(t)|+|V(t)|+|E(t)|+|I(t)|+|R(t)|\}. \end{array}$$

So 1 will become:


$$\begin{array}{cc} \psi_{1}(t,v(t))= & \Lambda +\tau V-(\beta I+\theta +\mu )S,\\ \psi_{2}(t,v(t))= & \theta S-\left((1-\epsilon )\beta I+\tau +\mu \right)V,\\ \psi_{3}(t,v(t))= & \beta IS+(1-\epsilon )\beta IV-(\delta +\mu )E,\\ \psi_{4}(t,v(t))= & \delta E-(\omega +d+\mu )I,\\ \psi_{5}(t,v(t))= & \omega I-\mu R. \end{array}$$ (12)

Now the compact form of the system will be


$$\begin{array}{c} \frac{dv}{dt}=\psi (t,v(t)),\;v(t)=(S(t),V(t),E(t),I(t),R(t){)}^{T}, \end{array}$$

where $\psi (t,v(t))=(\psi_{1}(t,v(t)),\psi_{2}(t,v(t)),\psi_{3}(t,v(t)),\psi_{4}(t,v(t)),\psi_{5}(t,v(t)){)}^{T}$.

The following integral equations can be obtained using eq. 12 and Lemma 2.5 in equation (1):


$$\begin{array}{c} S(t)=(S{)}_{0}+\frac{2(1-\zeta )}{M(\zeta )(2-\zeta )}(\psi_{1}(t,v(t))-\psi_{1}(0,v(0)))+\frac{2\zeta }{M(\zeta )(2-\zeta )}\int_{0}^{t}\psi_{1}(𝒦,v(𝒦))d𝒦,\\ V(t)=(V{)}_{0}+\frac{2(1-\zeta )}{M(\zeta )(2-\zeta )}(\psi_{2}(t,v(t))-\psi_{2}(0,v(0)))+\frac{2\zeta }{M(\zeta )(2-\zeta )}\int_{0}^{t}\psi_{2}(𝒦,v(𝒦))d𝒦,\\ E(t)=(E{)}_{0}+\frac{2(1-\zeta )}{M(\zeta )(2-\zeta )}(\psi_{3}(t,v(t))-\psi_{3}(0,v(0)))+\frac{2\zeta }{M(\zeta )(2-\zeta )}\int_{0}^{t}\psi_{3}(𝒦,v(𝒦))d𝒦,\\ I(t)=(I{)}_{0}+\frac{2(1-\zeta )}{M(\zeta )(2-\zeta )}(\psi_{4}(t,v(t))-\psi_{4}(0,v(0)))+\frac{2\zeta }{M(\zeta )(2-\zeta )}\int_{0}^{t}\psi_{4}(𝒦,v(𝒦))d𝒦,\\ R(t)=(R{)}_{0}+\frac{2(1-\zeta )}{M(\zeta )(2-\zeta )}(\psi_{5}(t,v(t))-\psi_{5}(0,v(0)))+\frac{2\zeta }{M(\zeta )(2-\zeta )}\int_{0}^{t}\psi_{5}(𝒦,v(𝒦))d𝒦, \end{array}$$

Denote


$$\begin{array}{cc} (w_{1}{)}_{0}= & ((S{)}_{0},(V{)}_{0},(E{)}_{0},(I{)}_{0},(R{)}_{0}),\\ w_{1}(t)= & (S(t),V(t),E(t),I(t),R(t)), \end{array}$$

and


$$\begin{array}{c} \psi (t,w_{1}(t))=(\psi_{1}(t,w_{1}(t)),\psi_{2}(t,w_{1}(t)),\psi_{3}(t,w_{1}(t)),\psi_{4}(t,w_{1}(t)),\psi_{5}(t,w_{1}(t))). \end{array}$$

Therefore, the above system of integral equations becomes:


$$\begin{array}{c} w_{1}(t)=(w_{1}{)}_{0}+\frac{2(1-\zeta )}{M(\zeta )(2-\zeta )}\psi (t,w_{1}(t)-(w_{1}{)}_{0})+\frac{2\zeta }{M(\zeta )(2-\zeta )}\int_{0}^{t}\psi (𝒦,v(𝒦))d𝒦, \end{array}$$ (13)

Let us take the hypothesis: $\left(\mu_{1}\right)$ There exist a constant λ∈(0,1) such that $|\psi (t,w_{1})-\psi (t,\bar{w_{1}})|\leq \lambda |w_{1}-\bar{w_{1}}|,\;\;H_{1}$ For all $w_{1},\bar{w_{1}}\in R$ and t∈[0,T]. (*H*_2_) There exist two positive functions $a_{1},b_{1}\in C[0,T]$ such that $|\psi (t,w_{1})\leq a_{1}(t)+b_{1}(t)||w_{1}|,$ for all $t\in [0,T],\;\;w_{1}\in R$

Denote


$$\begin{array}{c} M_{0}=\underset{t\in [0,T]}{sup}a_{1}(t),\\ M_{1}=\underset{t\in [0,T]}{sup}b_{1}(t). \end{array}$$

Now, two operators *L*_1_ and *L*_2_ can be defined using Equation 14.


$$\begin{array}{c} L_{1}w_{1}(t)=(w_{1}{)}_{0}+\frac{2(1-\zeta )}{M(\zeta )(2-\zeta )}\psi (t,w_{1}(t)-(w_{1}{)}_{0}) \end{array}$$ (14)

$$\begin{array}{c} L_{2}w_{1}(t)=\frac{2\zeta }{M(\zeta )(2-\zeta )}\int_{0}^{t}\psi (𝒦,v(𝒦))d𝒦, \end{array}$$ (15)

Now, we can state the main theorems of this research study.

**Theorem 3.1.** *The problem (1) has at least one solution if the hypotheses (H*_*1*_*) and (H*_*2*_*) hold and the following condition is satisfied:*


$$\begin{array}{c} \frac{2\lambda (1-\zeta )}{M(\zeta )(2-\zeta )}<1 \end{array}$$ (16)

*Proof.* We use Krasnoselskii’s fixed point theorem (Theorem 2.3) and need to satisfy all three of its assumptions. For the first assumption, we let


$$\begin{array}{c} {\tilde{X}}_{\gamma }=\{v\in \tilde{X}:\|v\|\leq \gamma ,\gamma >0\}. \end{array}$$

With


$$\begin{array}{c} \gamma \geq |(w_{1}{)}_{0}|\frac{2(1-\zeta )}{\left(2-\zeta )M(\zeta \right)}(2M_{0}+2M_{1}\gamma +M_{1}|(w_{1}{)}_{0}|) \end{array}$$ (17)

be a closed set of $\tilde{X}$ and convex also. Then, for $w_{1},\bar{w_{1}}\in {\tilde{X}}_{\gamma }$ we have


$$\begin{array}{c} \begin{array}{cc} \left|L_{1}w_{1}(t)-L_{1}\bar{w_{1}}(t)\right| & =(w_{1}{)}_{0}+\frac{2(1-\zeta )}{M(\zeta )(2-\zeta )}\psi (t,w_{1}(t)-(w_{1}{)}_{0})-[(w_{1}{)}_{0}+\frac{2\zeta }{M(\zeta )(2-\zeta )}\int_{0}^{t}\psi (t,\bar{w_{1}}(t)-(w_{1}{)}_{0})]\\ & \leq \frac{2(1-\zeta )}{M(\zeta )(2-\zeta )}|\psi (t,w_{1}(t)-(w_{1}{)}_{0})-\psi (t,\bar{w_{1}}(t)-(w_{1}{)}_{0})|\\ & \leq \frac{2(1-\zeta )\lambda }{M(\zeta )(2-\zeta )}|w_{1}(t)-\bar{w_{1}}(t)| \end{array} \end{array}$$

Hence,


$$\begin{array}{c} \left\|L_{1}w_{1}-L_{1}\bar{w_{1}}\|\leq \frac{2(1-\zeta )\lambda }{M(\zeta )(2-\zeta )}\|w_{1}-\bar{w_{1}}\right\| \end{array}$$ (18)

This result guarantees the contraction of the operator *L*_1_, and thus the second requirement of Theorem 2.3 is fulfilled.

Next, we demonstrate that *L*_2_ is uniformly bounded and equicontinuous.

Certainly, *L*_2_ is continuous, as is Ψ, and for all $w_{1}\in {\tilde{X}}_{\gamma }$ one has


$$\begin{array}{c} |L_{2}w_{1}(t)|\leq \frac{2(\zeta )}{M(\zeta )(2-\zeta )}|\int_{0}^{t}\psi (𝒦,w_{1}(𝒦))|d𝒦\leq \frac{2(\zeta )}{M(\zeta )(2-\zeta )}[M_{0}+M_{1}\gamma ]. \end{array}$$

So,


$$\begin{array}{c} \left\|L_{2}w_{1}(t)\|\leq \frac{2(\zeta )}{M(\zeta )(2-\zeta )}[M_{0}+M_{1}\gamma \right] \end{array}$$ (19)

Therefore, *L*_2_ is bounded uniformly. In order to examine the equicontinuity of *L*_2_, take $t_{1},t_{2}\in [0,T]$ with $w_{1}\in {\tilde{X}}_{\gamma }$, then


$$\begin{array}{cc} \left|L_{2}(w_{1})(t_{1})-L_{2}(w_{1})(t_{2})\right| & =\frac{2(\zeta )}{M(\zeta )(2-\zeta )}\left|\int_{0}^{t_{1}}\Psi (t,w_{1}(𝒦))\;d𝒦-\int_{0}^{t_{2}}\Psi (t,w_{1}(𝒦))\;d𝒦\right|\\ & \leq \frac{2(\zeta )}{M(\zeta )(2-\zeta )}\left|\int_{0}^{t_{2}}\Psi (𝒦,w_{1}(𝒦))\;d𝒦\right|\\ & \leq \frac{2(\zeta )}{M(\zeta )(2-\zeta )}(M_{0}+M_{1}\gamma )(t_{2}-t_{1}) \end{array}$$ (20)

As $t_{1}\to t_{2}$, the RHS of eq 19 approaches to zero. Therefore, *L*_2_ is equicontinuous.

Hence, *L*_2_ is relatively compact with the help of Arzela-Ascoli theorem.

Next, let $w_{1},z\in {\tilde{X}}_{\gamma }$, then


$$\begin{array}{cc} \left|L_{1}w_{1}(t)\right| & =|(w_{1}{)}_{0}|+\frac{2(1-\zeta )}{M(\zeta )(2-\zeta )}|\psi (t,w_{1}(t)-(w_{1}{)}_{0})|\\ & \leq |(w_{1}{)}_{0}|+\frac{2(1-\zeta )}{M(\zeta )(2-\zeta )}(M_{0}+M_{1}|w_{1}(t)-(w_{1}{)}_{0}|)\\ & \leq |(w_{1}{)}_{0}|+\frac{2(1-\zeta )}{M(\zeta )(2-\zeta )}(M_{0}+M_{1}(\gamma +|(w_{1}{)}_{0}|)) \end{array}$$

By using Eq. 16 and 18, we get


$$\begin{array}{cc} & \left|L_{1}w_{1}(t)-L_{2}z(t)|\leq |L_{1}w_{1}(t)|+|L_{2}z(t)\right|\\ & \leq |(w_{1}{)}_{0}|+\frac{2(1-\zeta )}{\left(2-\zeta )M(\zeta \right)}(2M_{0}+2M_{1}\gamma +M_{1}|(w_{1}{)}_{0}|)\leq \gamma . \end{array}$$

As all the hypotheses of Theorem 2.3 are satisfied, problem (1) possesses at least one solution.

Next, we address the uniqueness of this solution. □

**Theorem 3.2.** *If Hypotheses (H*_*1*_*) and (H*_*2*_*) hold and the subsequent inequality is satisfied:*


$$\begin{array}{c} \frac{2\lambda (1-\zeta +\zeta 𝒦)}{M(\zeta )(2-\zeta )}<1, \end{array}$$ (21)

*Then, the solution of the problem 1 will be unique.*

*Proof.* Now, *L*_3_ is defined on $\tilde{X}$ through


$$\begin{array}{c} \begin{array}{c} L_{3}(w_{1})(t)=(w_{1}{)}_{0}+\frac{2(1-\lambda )}{\left(2-\zeta )(\zeta -ℳ\right)}\Psi (t,w_{1}(t))-\frac{\Psi (0,(w_{1}{)}_{0})}{\left(2-\zeta )(\zeta -ℳ\right)}+\frac{2}{\left(2-\zeta )(\zeta -ℳ\right)}\int_{0}^{t}\Psi (𝒦,w_{1}(𝒦))d𝒦, \end{array} \end{array}$$ (22)

Let $(w_{1}),\tilde{w_{1}}\in \tilde{X}$, then


$$\begin{array}{cc} \left|L_{3}(w_{1})(t)-L_{3}(\tilde{w_{1}})(t)\right| & \leq \frac{2(1-\zeta )}{\left(2-\zeta )(M(\zeta )\right)}|\Psi (t,w_{1}(t))-\Psi (t,\tilde{w_{1}}(t))|\\ & +\frac{2\zeta }{\left(2-\zeta )(M(\zeta )\right)}\int_{0}^{t}|\Psi (𝒦,w_{1}(𝒦))-\Psi (𝒦,\tilde{w_{1}}(𝒦))|d𝒦,\\ & \leq \frac{2\lambda (1-\zeta +\zeta 𝒦)}{\left(2-\zeta )(M(\zeta )\right)}𝒦\|w_{1}-\tilde{w_{1}}\|, \end{array}$$

Hence


$$\begin{array}{c} \|L_{3}(w_{1})-L_{3}(\tilde{w_{1}})\|\leq \frac{2\lambda (1-\zeta +\zeta 𝒦)}{\left(2-\zeta )(M(\zeta )\right)}𝒦\|w_{1}-\tilde{w_{1}}\|. \end{array}$$ (23)

As, it has been found through eq. 23, that *L*_3_ is a contraction, so it is concluded from the principle of Banach’s contraction that the fixed point of *L*_3_ is unique and it is the unique solution of eq. 13. Consequently, the solution to Problem 1 will be unique as well. It can also be investigated through the use of another kind of fixed-point theorem that is labeled as the Fixed Point Theorem of Schauder. □

**Theorem 3.3.** *Let us consider that*


$$\begin{array}{c} 𝒦<(\frac{2(1-\zeta )M(\zeta )}{5max((\omega +𝒱),(𝒱+(1-\epsilon )\mu ),\left(\zeta_{1}+\omega \right),(𝒱+\zeta_{1}),\bar{\omega })}). \end{array}$$ (24)

*Then, there will be at least one solution to problem (1).*

*Proof.* Take


$$\begin{array}{c} (L_{3}{)}_{i}v(t)=v_{0}+\frac{2(1-\zeta )}{\left(2-\zeta )(M(\zeta )\right)}|\Psi_{i}(t,v(t))-\Psi_{i}(0,v(0))|+\frac{2\zeta }{\left(2-\zeta )(M(\zeta )\right)}\int_{0}^{t}\Psi_{i}(𝒦,v(𝒦))d𝒦, \end{array}$$

Then


$$\begin{array}{c} L_{3}v(t)=((L_{3}{)}_{1}v(t),(L_{3}{)}_{2}v(t),(L_{3}{)}_{3}v(t),(L_{3}{)}_{4}v(t),(L_{3}{)}_{5}v(t)). \end{array}$$

**(I)**

Operator *L*_3_ will be continuous, for the sequence $v_{n}=((S{)}_{n},(V{)}_{n},(E{)}_{n},(I{)}_{n},(R{)}_{n})$ such that $\lim_{n\to \infty }v_{n}=v$ we have, for all t∈[0,T].


$$\begin{array}{c} \begin{array}{cc} |(L_{3}{)}_{i}v_{n}(t)-(L_{3}{)}_{i}v(t)|= & v_{0}+\frac{2(1-\zeta )}{\left(2-\zeta )(M(\zeta )\right)}|\Psi_{i}(t,v_{n}(t))-\Psi_{i}(0,v(0))|+\frac{2\zeta }{\left(2-\zeta )(M(\zeta )\right)}\int_{0}^{t}\Psi_{i}(𝒦,v(𝒦)d𝒦\\ & \left[-v_{0}+\frac{2(1-\zeta )}{\left(2-\zeta )(M(\zeta )\right)}|\Psi_{i}(t,v(t))-\Psi_{i}(0,v(0))|+\frac{2\zeta }{\left(2-\zeta )(M(\zeta )\right)}\int_{0}^{t}\Psi_{i}(𝒦,v(𝒦))d𝒦\right]\\ & \left|\frac{2(1-\zeta )}{\left(2-\zeta )(M(\zeta )\right)}|\Psi_{i}(t,v_{n}(t))-\Psi_{i}(0,v(0))|+\frac{2\zeta }{\left(2-\zeta )(M(\zeta )\right)}\int_{0}^{t}\Psi_{i}(𝒦,v_{n}(𝒦))-v(𝒦)d𝒦\right|,\\ & \leq \frac{2(1-\zeta )}{\left(2-\zeta )(M(\zeta )\right)}|\Psi_{i}(t,v_{n}(t))-\Psi_{i}(0,v(0))|+\frac{2\zeta }{\left(2-\zeta )(M(\zeta )\right)}\int_{0}^{t}|\Psi_{i}(𝒦,v_{n}(𝒦))-v(𝒦)d𝒦|. \end{array} \end{array}$$ (25)

Eq. 12 is satisfied for each 1≤i≤5. This indicates that in $\psi_{i}(t,v_{n}(t))\to \psi_{i}(t,v(t))$ in $[0,T]\times R^{5}$. For $\psi_{1}$, we have


$$\begin{array}{c} \begin{array}{cc} \left|\psi_{1}(t,v_{n}(t))-\psi_{1}(t,v(t))\right| & =|\Lambda +\tau (V{)}_{n}-(\beta (I{)}_{n}+\theta +\mu )(S{)}_{n}\\ & \;-(\Lambda +\tau V-(\beta I+\theta +\mu )S)|,\\ & =|\tau ((V{)}_{n}-V)-\beta ((I{)}_{n}(S{)}_{n}-IS)-(\theta +\mu )((S{)}_{n}-S)|,\\ & \leq \tau |(V{)}_{n}-V|+\beta |(I{)}_{n}(S{)}_{n}-IS|+(\theta +\mu )|(S{)}_{n}-S|,\\ & \leq \tau \|v_{n}-v\|+\beta \left(\|(I{)}_{n}\|\;|(S{)}_{n}-S|+\|S\|\;|(I{)}_{n}-I|\right)+(\theta +\mu )\|v_{n}-v\|,\\ & \leq \left(\tau +\theta +\mu +\beta (\|(I{)}_{n}\|+\|S\|)\right)\|v_{n}-v\|. \end{array} \end{array}$$

Then,


$$\begin{array}{c} |(L_{3}{)}_{1}v_{n}(t)-(L_{3}{)}_{1}v(t)|\leq \frac{2(1-\zeta )}{\left(2-\zeta )M(\zeta \right)}+\frac{2\zeta 𝒦}{\left(2-\zeta )M(\zeta \right)}\{\omega +𝒱+\mu (\|(I{)}_{n}\|+\|S\|)\}\;\|v_{n}-v\|, \end{array}$$ (26)

which implies


$$\begin{array}{c} |(L_{3}{)}_{1}v_{n}(t)-(L_{3}{)}_{1}v(t)|\;⟶\;0,\;\text{as }n\to \infty . \end{array}$$

Similarly, for *i* = 1,2,3,4,5, we have


$$\begin{array}{c} |(L_{3}{)}_{i}v_{n}(t)-(L_{3}{)}_{i}v(t)|\;⟶\;0,\;\text{as }n\to \infty , \end{array}$$

and hence


$$\begin{array}{c} \lim_{n\to \infty }\|L_{3}v_{n}-L_{3}v\|=0. \end{array}$$

The continuity of *L*_3_ is established

**(II)**

Let $\tilde{X}$ has a subset *A* defined by


$$\begin{array}{c} A=\{v\in \tilde{X}:\;\|v\|\leq \xi \}, \end{array}$$

where ξ>0 will be chosen below. Use the sup norm on [0, *T*] as before. For the system defined in equation 1, the nonlinear components are:


$$\begin{array}{c} \begin{array}{cc} \psi_{1}(t,v) & =\Lambda +\tau V-(\beta I+\theta +\mu )S,\\ \psi_{2}(t,v) & =\theta S-((1-\epsilon )\beta I+\tau +\mu )V,\\ \psi_{3}(t,v) & =\beta IS+(1-\epsilon )\beta IV-(\delta +\mu )E,\\ \psi_{4}(t,v) & =\delta E-(\omega +d+\mu )I,\\ \psi_{5}(t,v) & =\omega I-\mu R. \end{array} \end{array}$$

Let


$$\begin{array}{c} \begin{array}{cc} M_{1} & =\max\{\tau +\theta +\mu ,\;\theta +\tau +\mu ,\;\delta +\mu ,\;\delta +\omega +d+\mu ,\;\omega +\mu \}\\ M_{2} & =\max\{\beta ,\;(1-\epsilon )\beta ,\;\beta +(1-\epsilon )\beta \}\;=\;(2-\epsilon )\beta . \end{array} \end{array}$$

If v∈A (so ‖v‖≤ξ), then for each t∈[0,T] we have the pointwise estimates


$$\begin{array}{c} \begin{array}{cc} \left|\psi_{1}(t,v(t))\right| & =|\Lambda +\tau V-(\beta I+\theta +\mu )S|\\ & \leq |\Lambda |+\tau \|V\|+(\beta \|I\|+\theta +\mu )\|S\|\\ & \leq |\Lambda |+(\tau +\theta +\mu )\xi +\beta \xi^{2},\\ \left[4pt]|\psi_{2}(t,v(t))\right| & =|\theta S-((1-\epsilon )\beta I+\tau +\mu )V|\\ & \leq \theta \|S\|+((1-\epsilon )\beta \|I\|+\tau +\mu )\|V\|\\ & \leq (\theta +\tau +\mu )\xi +(1-\epsilon )\beta \xi^{2},\\ \left[4pt]|\psi_{3}(t,v(t))\right| & =|\beta IS+(1-\epsilon )\beta IV-(\delta +\mu )E|\\ & \leq \beta \|I\|\;\|S\|+(1-\epsilon )\beta \|I\|\;\|V\|+(\delta +\mu )\|E\|\\ & \leq (\delta +\mu )\xi +(\beta +(1-\epsilon )\beta )\xi^{2},\\ \left[4pt]|\psi_{4}(t,v(t))\right| & =|\delta E-(\omega +d+\mu )I|\leq (\delta +\omega +d+\mu )\xi ,\\ \left[4pt]|\psi_{5}(t,v(t))\right| & =|\omega I-\mu R|\leq (\omega +\mu )\xi . \end{array} \end{array}$$

Hence, for every i=1,...,5 and every t∈[0,T],


$$\begin{array}{c} |\psi_{i}(t,v(t))|\leq |\Lambda |+M_{1}\;\xi +M_{2}\;\xi^{2}. \end{array}$$ (27)

Using the same form of the operator *L*_3_ and the fractional constants that appear elsewhere in your manuscript, we obtain for each *i*:


$$\begin{array}{c} |(L_{3}{)}_{i}v(t)|\leq \frac{10(1-\zeta +\zeta K)}{M(\zeta )(2-\zeta )}(|\Lambda |+M_{1}\xi +M_{2}\xi^{2}). \end{array}$$ (28)

Choose ξ>0 so that (equivalently)


$$\begin{array}{c} \frac{10(1-\zeta +\zeta K)}{M(\zeta )(2-\zeta )}(|\Lambda |+M_{1}\xi +M_{2}\xi^{2})\leq \frac{\xi }{5}. \end{array}$$ (29)

Then from (28) we get


$$\begin{array}{c} |(L_{3}{)}_{i}v(t)|\leq \frac{\xi }{5}\;(i=1,...,5), \end{array}$$ (30)

and therefore


$$\begin{array}{c} \|L_{3}v\|=\sum_{i=1}^{5}\|(L_{3}{)}_{i}v\|\leq \sum_{i=1}^{5}\frac{\xi }{5}=\xi . \end{array}$$

Consequently $L_{3}(A)\subset A$.

**(III)**

We show that *L*_3_(*A*) is relatively compact. For this, consider $t_{1},t_{2}\in [0,T],\;t_{1}<t_{2}$ and v∈A Then, ∀i=1,2,3,4,5. *we get*


$$\begin{array}{c} \begin{array}{cc} \left|(L_{3}{)}_{i}v(t_{2})-(L_{3}{)}_{i}v(t_{1})\right|= & |\left(v_{0}+\frac{2(1-\zeta )}{\left(2-\zeta )M(\zeta \right)}[\Psi_{i}(t_{2},v(t_{2}))-\Psi_{i}(0,v(0))]+\frac{2\zeta }{\left(2-\zeta )M(\zeta \right)}\int_{0}^{t_{2}}\Psi_{i}(𝒦,v(𝒦))d𝒦\right)\\ & -\left(v_{0}+\frac{2(1-\zeta )}{\left(2-\zeta )M(\zeta \right)}[\Psi_{i}(t_{1},v(t_{1}))-\Psi_{i}(0,v(0))]+\frac{2\zeta }{\left(2-\zeta )M(\zeta \right)}\int_{0}^{t_{1}}\Psi_{i}(𝒦,v(𝒦))d𝒦\right)|\\ & \leq \left|\frac{2(1-\zeta )}{\left(2-\zeta )M(\zeta \right)}[\Psi_{i}(t_{2},v(t_{2}))-\Psi_{i}(t_{1},v(t_{1}))]\right|\\ & +\frac{2\zeta }{\left(2-\zeta )M(\zeta \right)}\left|\int_{0}^{t_{2}}\Psi_{i}(𝒦,v(𝒦))d𝒦-\int_{0}^{t_{1}}\Psi_{i}(𝒦,v(𝒦))d𝒦\right|\\ & \leq \frac{2(1-\zeta )}{\left(2-\zeta )M(\zeta \right)}\lambda |v(t_{2})-v(t_{1})|\\ & +\frac{2(1-\zeta )}{\left(2-\zeta )M(\zeta \right)}\left(\max\{(\omega +𝒱),(𝒱+(1-\epsilon )\mu ),(\zeta_{1}+\omega ),(𝒱+\zeta_{1}),\bar{\omega }\}\xi +\mu \xi^{2}\right). \end{array} \end{array}$$ (31)

Let $t_{1}\to t_{2}\forall \;\;i=1,2,3,4,5$, then the R.H.S of the above inequality will become zero. Therefore, we find that *L*_3_ is relatively compact, special thanks to the Ascoli-Arzela theorem and steps (I) to (II). Hence, the complete requirements of Schauder’s fixed point theorem are met; there is at least one fixed point for the operator *L*_3_. It is a solution of the problem 1. A new hypothesis can now give a unique theorem. □

**Theorem 3.4.** *Consider the system of equations defined i*n equation 1, and l*et L*_*3*_ *be the associated operator obtained from the fractional integral representation of the system.*

*Assume that there exists R > 0 such that for all admissible solutions v(t) = (S(t),V(t),E(t),I(t),R(t)). and*


$$\begin{array}{c} \tilde{v}(t)=(\tilde{S}(t),\tilde{V}(t),\tilde{E}(t),\tilde{I}(t),\tilde{R}(t)),\;t\in [0,T]. \end{array}$$

*Assume that*


$$\begin{array}{c} \|v{\|}_{\infty }\leq R,\;\|\tilde{v}{\|}_{\infty }\leq R. \end{array}$$

*Define*


$$\begin{array}{c} \Gamma :=\max\left\{\tau +(\theta +\mu )+\beta R,\;\theta +(\tau +\mu )+(1-\epsilon )\beta R,\;\beta R+(\delta +\mu ),\;\delta +(\omega +d+\mu ),\;\omega +\mu \right\}. \end{array}$$

*If*


$$\begin{array}{c} \frac{10\;\lambda (1-\zeta +\zeta 𝒦)}{M(\zeta )(2-\zeta )}\;\Gamma <1, \end{array}$$ (32)

*then there is a unique solution of the fractional problem on [0,T].*

*Proof.* Let *v* = (*S*,*V*,*E*,*I*,*R*) and $\tilde{v}=(\tilde{S},\tilde{V},\tilde{E},\tilde{I},\tilde{R})$ be two solutions in the solution space with $\|v{\|}_{\infty },\|\tilde{v}{\|}_{\infty }\leq R$. Denote the right-hand sides by $\psi_{1},...,\psi_{5}$:


$$\begin{array}{c} \begin{array}{cc} \psi_{1}(t,v) & =\Lambda +\tau V-(\beta I+\theta +\mu )S,\\ \psi_{2}(t,v) & =\theta S-((1-\epsilon )\beta I+\tau +\mu )V,\\ \psi_{3}(t,v) & =\beta IS+(1-\epsilon )\beta IV-(\delta +\mu )E,\\ \psi_{4}(t,v) & =\delta E-(\omega +d+\mu )I,\\ \psi_{5}(t,v) & =\omega I-\mu R. \end{array} \end{array}$$

We estimate the difference term by term:

**1. For** $\psi_{1}$:

$$\begin{array}{c} \begin{array}{cc} \left|\psi_{1}(t,v)-\psi_{1}(t,\tilde{v})\right| & \leq \tau |V-\tilde{V}|+\beta (|I||S-\tilde{S}|+|\tilde{S}||I-\tilde{I}|)+(\theta +\mu )|S-\tilde{S}|\\ & \leq (\tau +(\theta +\mu )+\beta R)\|v-\tilde{v}\|. \end{array} \end{array}$$

**2. For** $\psi_{2}$:

$$\begin{array}{c} |\psi_{2}(t,v)-\psi_{2}(t,\tilde{v})|\leq (\theta +(\tau +\mu )+(1-\epsilon )\beta R)\|v-\tilde{v}\|. \end{array}$$

**3. For** $\psi_{3}$:

$$\begin{array}{c} |\psi_{3}(t,v)-\psi_{3}(t,\tilde{v})|\leq (\beta R+(\delta +\mu ))\|v-\tilde{v}\|. \end{array}$$

**4. For** $\psi_{4}$:

$$\begin{array}{c} |\psi_{4}(t,v)-\psi_{4}(t,\tilde{v})|\leq (\delta +(\omega +d+\mu ))\|v-\tilde{v}\|. \end{array}$$

**5. For** $\psi_{5}$:

$$\begin{array}{c} |\psi_{5}(t,v)-\psi_{5}(t,\tilde{v})|\leq (\omega +\mu )\|v-\tilde{v}\|. \end{array}$$

Thus, for all i=1,...,5 we have


$$\begin{array}{c} |\psi_{i}(t,v)-\psi_{i}(t,\tilde{v})|\leq \Gamma \|v-\tilde{v}\|. \end{array}$$

Applying the fractional integral representation for *L*_3_, we get


$$\begin{array}{c} \|L_{3}v-L_{3}\tilde{v}\|\leq \frac{10\;\lambda (1-\zeta +\zeta 𝒦)}{M(\zeta )(2-\zeta )}\;\Gamma \;\|v-\tilde{v}\|. \end{array}$$

Under condition (32), the multiplicative constant is strictly less than 1, so *L*_3_ is a contraction on the solution space. Hence, with the help of the Banach contraction principle, it is concluded that the fixed point of *L*_3_ is unique, the problem’s unique solution on [0,*T*].

**Definition 3.1.** Let ϵ>0. The fractional-order problem (1) is said to be *Ulam–Hyers stable* if, for every function $w_{1}\in C(J,\mathbb{R})$ satisfying


$$\begin{array}{c} \left|{\;}^{CF}D_{0^{+}}^{\zeta }w_{1}(t)-\psi (t,w_{1}(t))\right|\leq \epsilon ,\;t\in J, \end{array}$$ (33)

there exists a unique solution z of problem (1) such that


$$\begin{array}{c} \|w_{1}-z\|\leq C\epsilon \end{array}$$

where C > 0 is a positive constant independent of ϵ.

**Remark 3.5.** *If inequality in theorem35 holds then there exist a function* ℵ *depending on* $w_{1}\in \tilde{X}$ *such that* ℵ(0)=0,|ℵ(t)|≤ϵ *for all* t∈[0,T] *and*


$$\begin{array}{c} {\;}^{CF}D_{0^{+}}^{\zeta }\left(w_{1}(t)\right)=\psi (t,w_{1}(t))+ℵ(t),\;\;t\in [0,T]. \end{array}$$ (34)

**Lemma 3.6.** *The solution of the problem*


$$\begin{array}{c} {\;}^{CF}D_{0^{+}}^{\zeta }\left(w_{1}(t)\right)=\psi (t,w_{1}(t))+ℵ(t),\;\;t\in [0,T], \end{array}$$ (35)

$$\begin{array}{c} w_{1}(0)=0, \end{array}$$ (36)

*Is*


$$\begin{array}{cc} w_{1}(t)= & (w_{1}{)}_{0}+\frac{2(1-\zeta )}{M(\zeta )(2-\zeta )}(\psi (t,w_{1}(t)-\psi (0,(w_{1}{)}_{0}))+\frac{2\zeta }{M(\zeta )(2-\zeta )}\int_{0}^{t}\psi (𝒦,w_{1}(𝒦))d𝒦\\ & +\frac{2(1-\zeta )}{M(\zeta )(2-\zeta )}ℵ(t)+\frac{2\zeta }{M(\zeta )(2-\zeta )}\int_{0}^{t}ℵ(t)d𝒦, \end{array}$$

*In addition, the following inequality is satisfied by the solution*


$$\begin{array}{cc} w_{1}(t)- & \left[(w_{1}{)}_{0}+\frac{2(1-\zeta )}{M(\zeta )(2-\zeta )}\psi (t,w_{1}(t)-\psi (0,(w_{1}{)}_{0}))+\frac{2\zeta }{M(\zeta )(2-\zeta )}\int_{0}^{t}\psi (𝒦,w_{1}(𝒦))d𝒦\right]\\ \leq & \frac{2\lambda (1-\zeta +\zeta 𝒦)}{M(\zeta )(2-\zeta )}\epsilon . \end{array}$$ (37)

**Theorem 3.7.** *If the hypothesis H*_*1*_ *and H*_*2*_ *are satisfies and the following inequality exist:*


$$\begin{array}{c} \frac{\left(2-\zeta +\zeta 𝒦\right)M(\zeta )}{M(\zeta )(2-\zeta )-2\delta (1-\zeta +\zeta 𝒦)}<1, \end{array}$$ (38)

*then, The problem 1 is Ulam-Hyers stable.*

*Proof.* Assume v is a solution of inequality 2.30 and problem (1) has solution $\tilde{v}$, then we get


$$\begin{array}{c} \begin{array}{cc} \|v-v_{0}\|=\;\; & \underset{t\in [0,T]}{max}|v(t)+\frac{2(1-\zeta )}{M(\zeta )(2-\zeta )}\psi (t,\tilde{v}-\psi (0,{\tilde{v}}_{0}))+\frac{2\zeta }{M(\zeta )(2-\zeta )}\int_{0}^{t}\psi (𝒦,\tilde{v}(𝒦))d𝒦|,\\ & \leq \underset{t\in [0,T]}{max}|v-[v_{0}+\frac{2(1-\zeta )}{M(\zeta )(2-\zeta )}\psi (t,v-\psi (0,v_{0}))+\frac{2\eta }{M(\zeta )(2-\zeta )}\int_{0}^{t}\psi (𝒦,v(𝒦))d𝒦]|,\\ & +\frac{2(1-\zeta )}{M(\zeta )(2-\zeta )}\underset{t\in [0,T]}{max}\{|\psi (t,v-\psi (0,\tilde{v}))\}+\frac{2\zeta }{M(\zeta )(2-\zeta )}\underset{t\in [0,T]}{max}\{|\int_{0}^{t}(\psi (𝒦,v(𝒦))-\psi (𝒦,\tilde{v}(𝒦))d𝒦)|\}, \end{array} \end{array}$$

Since


$$\begin{array}{c} |\Psi (t,v(t))-\Psi (t,\tilde{v}(t))|\leq \|v(t)-\tilde{v}(t)\|, \end{array}$$

we get


$$\begin{array}{c} \|v(t)-\tilde{v}(t)\|\leq \frac{2(1-\zeta +\zeta 𝒦)}{M(\zeta )(2-\zeta )}\epsilon +\frac{2\delta (1-\zeta +\zeta 𝒦)}{M(\zeta )(2-\zeta )}\|v(t)-\tilde{v}(t)\|, \end{array}$$ (39)

From eq. 39 we get


$$\begin{array}{c} \|v(t)-\tilde{v}(t)\|\leq \frac{\left(2-\zeta +\zeta 𝒦\right)M(\zeta )}{M(\zeta )(2-\zeta )-2\delta (1-\zeta +\zeta 𝒦)}\epsilon . \end{array}$$ (40)

From inequality eq. 40, it is concluded that the Ulam-Hyers stable is the solution of problem 1. □

## 5. Implimentation of three-step Adams-Bashforth scheme

Numerous novel analytical techniques have been developed recently to address the vast array of nonlinear fractional derivative models that were applied as problem models in the actual world. The homotopy analysis transform method (HASTM) and the homotopy analysis Sumudu transform technique (HASTM) [34] are two among the new analytical techniques [35]. and the Laplace transform method for local fractional homotopy perturbation (LFHPLTM) [36]. Furthermore, a variety of numerical techniques have been advanced to yield approximations for fractional differential equation solutions. These techniques, which usually involve discretizing the independent variable, include adaptations of integer-order techniques like the finite difference [37], finite element [38], and Adams–Bashforth–Moulton type predictor-corrector methods [39]. In this paper, we will solve the Caputo–Fabrizio fractional model 12 numerically using the three–step fractional Adams–Bashforth method. Firstly, this section calls for the three-step fractional Adams–Bashforth scheme to solve the fractional HIV/AIDS model in Eqs. 12 numerically, for a range of reasonable parameter values and fractional orders. Let us take the differential form of the Caputo-Fabrizio fractional equation


$$\begin{array}{c} {\;}^{CF}D_{t}^{\zeta }\left(v(t)\right)=f_{1}(t,v(t)),\;\;\;\;0<\zeta <1, \end{array}$$ (41)

By eq. 5, ${\;}^{CF}D_{t}^{\zeta }$ is Caputo-Fabrizio fractional derivative


$$\begin{array}{c} {\;}^{CF}I_{t}^{\zeta }\left(f_{1}(t)\right)=\frac{1-\zeta }{M(\zeta )}f_{1}(t)+\frac{\zeta }{M(\zeta )}\int_{0}^{t}f_{1}(x)dx. \end{array}$$ (42)

Applying the above integral equation to both sides of Eq. 41, we get


$$\begin{array}{cc} {\;}^{CF}I_{t}^{\zeta }{\;}^{CF}D_{t}^{\zeta }\left(v(t)\right)= & {\;}^{CF}I_{t}^{\zeta }\left(f_{1}(t,v(t))\right),\\ v(t)-v(0)= & {\;}^{CF}I_{t}^{\zeta }\left(f_{1}(t,v(t))\right),\\ = & \frac{1-\zeta }{M(\zeta )}\left(f_{1}(t,v(t))\right)+\frac{\zeta }{M(\zeta )}\int_{0}^{t}\left(f_{1}(s,v(s))\right)ds. \end{array}$$ (43)

The Following sequence $t_{0}=0,\;\;t_{k}+1=t_{k}+h,k=0,1,2,...,n--1,t_{n}=t$ can be obtained by categorizing the time interval [0,*t*] into steps of *h*. The following recursive formulas can be construc*t*ed using Eq. 43:


$$\begin{array}{c} v(t_{k+1})-v(0)=\frac{1-\zeta }{M(\zeta )}\left(f_{1}(t_{k},v(t_{k}))\right)+\frac{\zeta }{M(\zeta )}\int_{0}^{t_{k+1}}\left(f_{1}(t,v(t))\right)dt. \end{array}$$ (44)

and


$$\begin{array}{c} v(t_{k})-v(0)=\frac{1-\zeta }{M(\zeta )}\left(f_{1}(t_{k-1},v(t_{k-1}))\right)+\frac{\zeta }{M(\zeta )}\int_{0}^{t_{k}}\left(f_{1}(t,v(t))\right)dt. \end{array}$$ (45)

Subtracting Eq. 45 from Eq. 44, we obtain


$$\begin{array}{c} v(t_{k+1})-v(t_{k})=\frac{1-\zeta }{M(\zeta )}[\left(f_{1}(t_{k},v(t_{k}))\right)-\left(f_{1}(t_{k-1},v(t_{k-1}))\right)]+\frac{\zeta }{M(\zeta )}\int_{t_{k}}^{t_{k+1}}\left(f_{1}(t,v(t))\right)dt. \end{array}$$ (46)

Now, by approximating the integral $\int_{t_{k}}^{t_{k+1}}\left(f_{1}(t,v(t))\right)dt$ in the above equation by using $\int_{t_{k}}^{t_{k+1}}P_{2}(t)dt$,the Adams-Bashforth-type three-step formula is derived. consider *P*_2_(*t*) second degree Lagrange interpolating polynomial from the set of given points

$\left(t_{k-2},f_{1}(t_{k-2},v(t_{k-2})),(t_{k-1},f_{1}(t_{k-1},v(t_{k-1}))),(t_{k},f_{1}(t_{k},v(t_{k})))\right)$. That is,


$$\begin{array}{c} P_{2}(t)=\sum_{i=0}^{2}f_{1}(t_{k-i},v_{k-i})L_{i}(t), \end{array}$$ (47)

Here $L_{i}(t)$ represents the Lagrange basis polynomials on the three points $\left(t_{k--2},t_{k--1},t_{k}\right)$. Using the change of variable $\frac{t_{k+1}-t}{h}$, using for Lagrange basis polynomials and integrating that results in


$$\begin{array}{c} \begin{array}{cc} \int_{t_{k}}^{t_{k+1}}f_{1}(t,v(t))dt & =h\int_{0}^{1}[\frac{\left(s-2)(s-3\right)}{\left(1-2)(1-3\right)}f_{1}(t_{k},v_{k})+\frac{\left(s-1)(s-3\right)}{\left(2-1)(2-3\right)}f_{1}(t_{k-1},v_{k-1})+\frac{\left(s-1)(s-2\right)}{\left(3-2)(3-1\right)}f_{1}(t_{k-2},v_{k-2})]\\ & =h[\frac{23}{12}f_{1}(t_{k},v_{k})-\frac{4}{3}f_{1}(t_{k-1},v_{k-1})+\frac{5}{12}f_{1}(t_{k-2},v_{k-2})], \end{array} \end{array}$$ (48)

where $v_{k-2}=v(t_{k-2}),v_{k-1}=v(t_{k-1}),andv_{k}=v(t_{k})$ Then, inserting Eq. 48 into Eq. 46, we get the following iterative formula:


$$\begin{array}{c} \begin{array}{c} v_{k+1}=v_{k}+\frac{1}{M(\zeta )}[(1-\zeta )+\frac{23h\zeta }{12}]f_{1}(t_{k},v_{k})-\frac{1}{M(\zeta )}[(1-\zeta )+\frac{4h\zeta }{3}]f_{1}(t_{k-1},v_{k-1})\\ +[\frac{5h\zeta }{12M(\zeta )}]f_{1}(t_{k-2},v_{k-2}), \end{array} \end{array}$$ (49)

If we take as a special case ζ=1, Eq. 49 is reduced to the classical Adams–Bashforth three-step predictor formula.

The truncation error can be estimated for the three-step formula by the use of the error estimate for the Lagrange interpolating polynomial, that is


$$\begin{array}{cc} f_{1}(t,v_{t})= & P_{2}(t)+E_{2}(t),\\ E_{2}(t)= & \frac{f_{1}^{\left(3\right)}(\xi_{k},v(\xi_{k}))}{3!}(t-t_{k})(t-t_{k-1})(t-t_{k-2}),\;\;\;\xi_{k}\in (t_{k-2},t_{k}). \end{array}$$ (50)

Then, we have


$$\begin{array}{cc} \int_{t_{k}}^{t_{k+1}}E_{2}(t)dt= & \int_{t_{k}}^{t_{k+1}}\frac{f_{1}^{\left(3\right)}(\xi_{k},v(\xi_{k}))}{3!}(t-t_{k})(t-t_{k-1})(t-t_{k-2})dt,\\ ≅ & -\frac{h^{4}f_{1}^{3}(\xi_{k},v(\xi_{k}))}{6(3!)}\int_{0}^{1}(s-1)(s-2)(s-3)ds,\\ = & \frac{3}{8}h^{4}f_{1}^{3}(\xi_{k},v(\xi_{k})). \end{array}$$ (51)

where $t_{k}\in (t_{k--2},t_{k+1})$, and this integral is approximated by using a mean value theorem. The right-hand side of Eq. 49 can be represented by $v_{k}$, then we get $v_{k+1}=v_{k}+\frac{3}{8}h^{4}f_{1}^{3}(\xi_{k},v(\xi_{k}))$. Therefore, with the help of Eq. 49, the local truncation error is obtained by


$$\begin{array}{cc} \tau_{k+1} & =\frac{\zeta }{M(\zeta )}\frac{\frac{3}{8}h^{4}f_{1}^{\left(3\right)}(\xi_{k},v(\xi_{k}))}{h}\\ & =\frac{3\zeta }{8M(\zeta )}h^{3}f_{1}^{\left(3\right)}(\xi_{k},v(\xi_{k})). \end{array}$$ (52)

Furthermore, to derive numerical solutions of the fractional model 12, the three-step fractional Adams–Bashforth scheme is used in Eq. 49. To get the numerical results, it is assumed that the fractional derivatives in system 12 follow the same order, $i.e.,\zeta_{1}=\zeta_{2}=\zeta_{3}=\zeta_{4}=\zeta_{5}=\zeta .$ The system can be written in vector form


$$\begin{array}{c} {\;}^{CF}D_{t}^{\zeta }\left(v(t)\right)=f_{1}(t,v(t)),\;\;\;\;0<\zeta <1, \end{array}$$ (53)

where


$$\begin{array}{c} v(t)=[\begin{array}{c} S(t)\\ V(t)\\ E(t)\\ I(t)\\ R(t) \end{array}],\;\;f_{1}(t,v(t))=[\begin{array}{c} \left(f_{1}{)}_{1}(t,v(t)\right)\\ \left(f_{1}{)}_{2}(t,v(t)\right)\\ \left(f_{1}{)}_{3}(t,v(t)\right)\\ \left(f_{1}{)}_{4}(t,v(t)\right)\\ \left(f_{1}{)}_{5}(t,v(t)\right) \end{array}]. \end{array}$$ (54)

Now, $f_{i},i=1,2,3,...,5,$, the scalar functions, can be defined from the system 12, $i.e.,(f_{1}{)}_{1}(t,v(t))=\Lambda +\tau V-(\beta I+\theta +\mu )S,(f_{1}{)}_{2}(t,v(t))$$=\theta S-\left((1-\epsilon )\beta I+\tau +\mu \right)V,(f_{1}{)}_{3}(t,v(t))\beta IS+(1-\epsilon )\beta IV-(\delta +\mu )E,(f_{1}{)}_{4}(t,v(t))=\delta E-(\omega +d+\mu )I;and\;(f_{1}{)}_{5}(t,v(t))=\omega I-\mu R.$

Applying the fractional integration to both sides of Eq. 52 we have


$$\begin{array}{cc} v(t)-v(0)= & {\;}^{CF}I_{t}^{\zeta }\left(f_{1}(t)\right),\\ = & \frac{1-\zeta }{M(\zeta )}\left(f_{1}(t,v(t))\right)+\frac{\zeta }{M(\zeta )}\int_{0}^{t}\left(f_{1}(s,v(s))\right)ds. \end{array}$$ (55)

Finally, the following Iterative formula is obtained.


$$\begin{array}{c} \begin{array}{c} v_{k+1}=v_{k}+\frac{1}{M(\zeta )}[(1-\zeta )+\frac{23h\zeta }{12}]f_{1}(t_{k},v_{k})-\frac{1}{M(\zeta )}[(1-\zeta )+\frac{4h\zeta }{3}]f_{1}(t_{k-1},v_{k-1})+[\frac{5h\zeta }{12M(\zeta )}]f_{1}(t_{k-2},v_{k-2}), \end{array} \end{array}$$ (56)

where $v_{k+1}=v(t_{k+1}),v_{k}=v(t_{k}),v_{k-1}=v(t_{k-1}),v_{k-2}=v(t_{k-2}),$

and $v_{0}=v(t_{0})=[S(t_{0}),I(t_{0}),A_{a}(t_{0}),I(t_{0}),R(t_{0}){]}^{T}.$

## 6. Deep neural network approximation of the norovirus model

Deep Neural Networks (DNNs) provide a flexible framework for solving systems of nonlinear ordinary differential equations without requiring discretization of the time domain. In this study, we consider a feedforward deep neural network to approximate the solution of the SVEIR norovirus model to achieve high accuracy in solving nonlinear systems. The neural network consists of multiple hidden layers, each with several neurons, as shown in Fig 2. The network takes the time variable *t* as input and outputs the predicted compartments:


$$\begin{array}{c} \hat{v}(t)=(\hat{S}(t),\hat{V}(t),\hat{E}(t),\hat{I}(t),\hat{R}(t)). \end{array}$$

**Fig 2:**
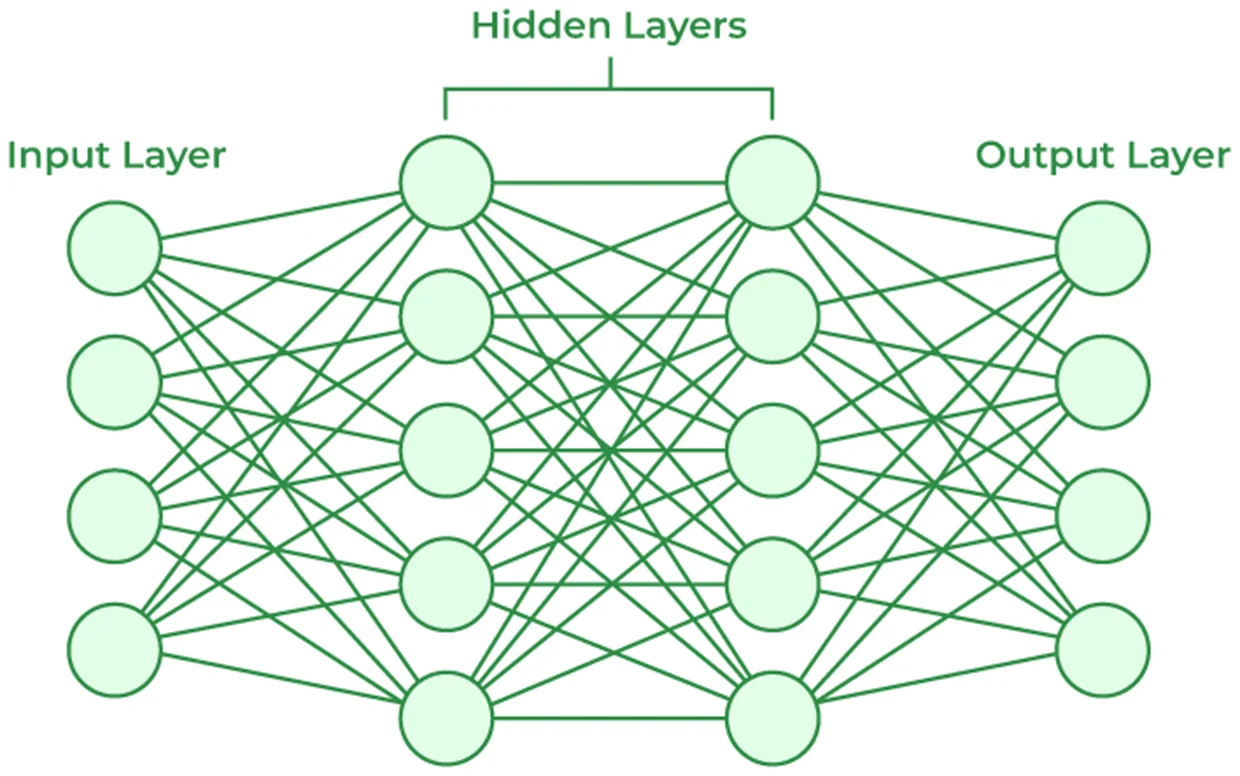
Deep Neural Network Architecture with multiple layers.

### 6.1 Activation function: NGNDG-AF

An activation function is a mathematical function used in neural networks to introduce nonlinearity. Without it, a neural network would behave like a simple linear model, limiting its ability to learn complex patterns. We propose a novel family of activation functions, known as NGNDG-AF [40], as defined in Equation (57).

NGNDG-AF distinguishes itself from other activation functions such as ReLU and ELU, despite certain similarities, by effectively capturing small negative input values, smoothness, and approximating continuous functions, thereby enhancing the model’s sensitivity. Its unbounded behavior in the positive direction facilitates faster training, while its lower-bounded nature acts as a form of regularization by limiting outputs within a controlled range instead of extending toward negative infinity. Collectively, these characteristics make NGNDG-AF a versatile activation function with strong potential to improve overall neural network performance.


$$\begin{array}{c} f\left(x\right)=\{\begin{array}{cc} g\left(x\right) & :\;\;\;x\geq 0\\ h\left(x\right) & :\;\;x<0 \end{array} \end{array}$$ (57)

In the NGNDG-AF framework, we consider two functions defined as g(x)=βx and $h(x)=\frac{\beta x}{1+x^{2}}$, where β is a constant parameter. The parameter β plays a key role in determining the sign and magnitude of the output, particularly for negative input values. The term βx governs the polarity of the response when the input is negative. Specifically, when β>0, the function *h*(*x*) yields negative values for negative inputs, whereas for β<0, it produces positive values. This behavior highlights the influence of β in shaping the activation response.


$$\begin{array}{c} f\left(x\right)=\{\begin{array}{cc} \beta x & :\;\;\;x\geq 0\\ \frac{\beta x}{1+x^{2}} & :\;\;x<0 \end{array} \end{array}$$ (58)

$$\begin{array}{c} f^{\prime }\left(x\right)=\{\begin{array}{cc} 1 & :\;\;\;x\geq 0\\ \frac{f\left(x\right)}{\beta x}-\frac{2{\left(f\left(x\right)\right)}^{2}}{\beta } & :\;\;x<0 \end{array} \end{array}$$ (59)

Fig 3 presents the graphical behavior of the NGNDG-AF activation function for both positive and negative values of the parameter β. The plot demonstrates how variations in β influence the function’s response across different input ranges. In particular, it highlights the changes in activation behavior for both positive and negative inputs as β varies. This adaptability reflects the function’s versatility, allowing it to exhibit behavior similar to ReLU under certain conditions while also enabling more complex activation patterns for other parameter choices.

**Fig 3:**
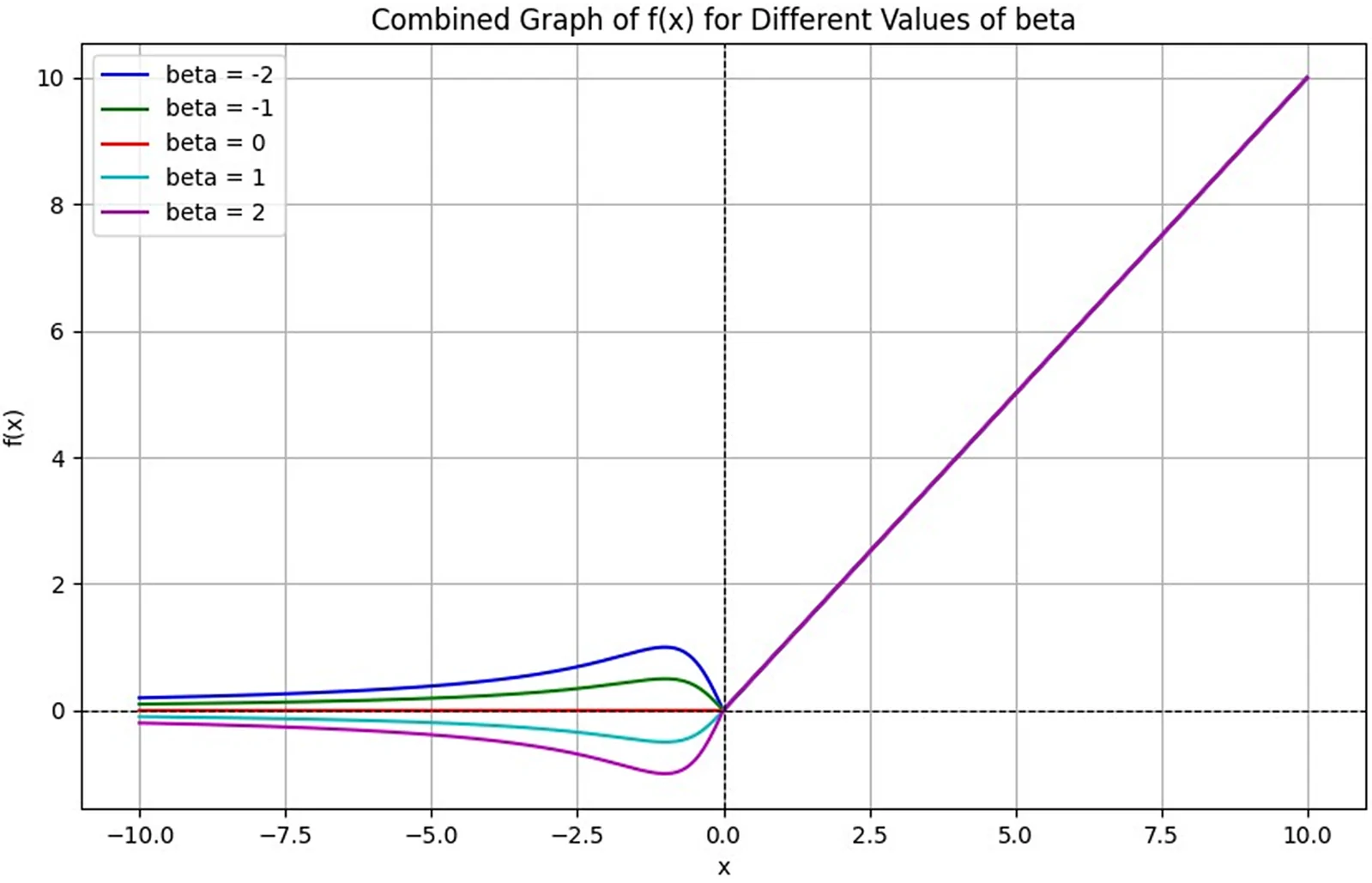
Plot of NGNDG-AF for different values of β.

### 6.2. Network architecture and forward propagation

The network is fully connected (dense) with multiple hidden layers. The input layer represents time *t*, while the output layer is associated with five compartments of the model. For training, *N* points are sampled from the domain Ω, forming the input matrix


$$\begin{array}{c} X=[\begin{array}{c} t_{1}\\ t_{2}\\ \vdots \\ t_{N} \end{array}]\in {\mathbb{R}}^{N\times 1}, \end{array}$$

and the corresponding network outputs are


$$\begin{array}{c} \hat{U}=[\begin{array}{ccccc} \hat{S}(t_{1}) & \hat{V}(t_{1}) & \hat{E}(t_{1}) & \hat{I}(t_{1}) & \hat{R}(t_{1})\\ \vdots & \vdots & \vdots & \vdots & \vdots \\ \hat{S}(t_{N}) & \hat{V}(t_{N}) & \hat{E}(t_{N}) & \hat{I}(t_{N}) & \hat{R}(t_{N}) \end{array}]\in {\mathbb{R}}^{N\times 5}. \end{array}$$

The general form of forward propagation for a network with *L* hidden layers can be defined as:


$$\begin{array}{cc} Z^{\left[l\right]} & =W^{\left[l\right]}A^{\left[l-1\right]}+b^{\left[l\right]},\;l=1,2,...,L,\\ A^{\left[l\right]} & =f(Z^{\left[l\right]}),\\ \hat{u}(t) & =W^{\left[L+1\right]}A^{\left[L\right]}+b^{\left[L+1\right]}, \end{array}$$ (60)

where *A*^[0]^ = *t*, *W*^[*l*]^ and *b*^[*l*]^ are the weights and biases of layer *l*, *f* is a nonlinear activation function (here NGNDG), and *Z*^[*l*]^, *A*^[*l*]^ are the pre-activation and activation vectors. By using the general form the forward propagation process through some hidden and output layers of the neural network can be mathematically expressed as follows:


$$\begin{array}{cc} Z^{\left[1\right]} & =W^{\left[1\right]}t+b^{\left[1\right]},\;A^{\left[1\right]}=f(Z^{\left[1\right]}), \end{array}$$ (61)

$$\begin{array}{cc} Z^{\left[2\right]} & =W^{\left[2\right]}A^{\left[1\right]}+b^{\left[2\right]},\;A^{\left[2\right]}=f(Z^{\left[2\right]}), \end{array}$$ (62)

$$\begin{array}{cc} Z^{\left[3\right]} & =W^{\left[3\right]}A^{\left[2\right]}+b^{\left[3\right]},\;A^{\left[3\right]}=f(Z^{\left[3\right]}), \end{array}$$ (63)

$$\begin{array}{cc} \hat{u}(t) & =W^{\left[4\right]}A^{\left[3\right]}+b^{\left[4\right]}. \end{array}$$ (64)

### 6.3. ODE residual and loss function

The residuals of the system of equations are defined as:


$$\begin{array}{cc} f_{1} & =\frac{d\hat{S}}{dt}-\left[\Lambda +\tau \hat{V}-(\beta \hat{I}+\theta +\mu )\hat{S}\right],\\ f_{2} & =\frac{d\hat{V}}{dt}-\left[\theta \hat{S}-((1-\epsilon )\beta \hat{I}+\tau +\mu )\hat{V}\right],\\ f_{3} & =\frac{d\hat{E}}{dt}-\left[\beta \hat{I}\hat{S}+(1-\epsilon )\beta \hat{I}\hat{V}-(\delta +\mu )\hat{E}\right],\\ f_{4} & =\frac{d\hat{I}}{dt}-\left[\delta \hat{E}-(\omega +d+\mu )\hat{I}\right],\\ f_{5} & =\frac{d\hat{R}}{dt}-\left[\omega \hat{I}-\mu \hat{R}\right]. \end{array}$$ (65)

We define the trial solution for each compartment to satisfy initial conditions:


$$\begin{array}{c} u_{j}(t;\theta )=g_{j}(t)+F_{j}(t)\;{\hat{u}}_{j}(t;\theta ),\;j=S,V,E,I,R, \end{array}$$ (66)

where $g_{j}(t)$ satisfies the initial condition and $F_{j}(t)$ is constructed such that the initial conditions are enforced automatically.

The network is trained to minimize the ODE residuals via the cost function:


$$\begin{array}{c} ℒ(\theta )=\frac{1}{N}\sum_{i=1}^{N}\sum_{j\in \{S,V,E,I,R\}}|{ℱ}_{j}(t_{i},u_{S},...,u_{R},u_{S}^{\prime },...,u_{R}^{\prime }){|}^{2}, \end{array}$$ (67)

where ${ℱ}_{j}$ denotes the residual of the *j*-th differential equation evaluated at the trial solution. Automatic differentiation is used to compute derivatives $u_{j}^{\prime }=du_{j}/dt$. Minimization of ℒ(θ) ensures that the network approximates the solution of the SVEIR system accurately. The learning process is unsupervised, as no exact solutions are required; the network learns purely from the ODE structure and initial conditions. Moreover, the neural network is trained using the Adam optimizer with a learning rate of 10^–2^. The training process is carried out for a sufficient number of epochs to ensure convergence. Automatic differentiation is employed to compute the derivatives of the neural network outputs with respect to time, enabling accurate evaluation of the ODE residuals. The performance of the proposed DNN model is evaluated by comparing its predictions with those obtained using a classical numerical method, which is discussed in a subsequent section.

## 7. Numerical simulation and discussion with deep neural network

We have implemented a deep neural network (DNN) to validate and compare the results obtained from the proposed mathematical model. The ODEs’ residual and initial conditions are incorporated into the loss function, and the network parameters are optimized through a gradient-based learning process. The model training is conducted over 1000 epochs. To achieve the best set of weights with minimal residual errors, we applied the Bayesian Regularization Scheme. The accuracy of the model is demonstrated in the accompanying figures.

Fig 4 presents the deep neural network (DNN) results for the susceptible compartment (S) under five different scenarios ranging from *S*_0_ to *S*_4_. Fig 4(a) shows the regression analysis for the training, testing, validation, and overall datasets. The regression coefficient is approximately equal to one, indicating an excellent correlation between the network outputs and the target values and confirming the high predictive accuracy of the proposed DNN model. Figs 4(b–f) illustrate the function fitting, error histogram, and training performance for the five scenarios. All networks were trained for 1000 epochs, and the validation checks remained zero throughout the training process, indicating stable convergence without early stopping. The best Mean Squared Error (MSE) values obtained for *S*_0_ to *S*_4_ are 543.8996, 584.2152, 665.8776, 740.3281, and 697.4525, respectively. Although the MSE values vary across the scenarios, they remain within an acceptable range, demonstrating the robustness and consistency of the proposed DNN in approximating the fractional-order susceptible dynamics. The corresponding optimization parameters also indicate satisfactory convergence. The gradients for the five cases are 88.1798, 169.9654, 147.8470, 89.9685, and 76.3109, respectively, while the damping parameter μ was automatically adjusted during the training process to improve convergence. The parameter norms and sums of squared parameters remained finite throughout training, further confirming the numerical stability of the network optimization. Fig 4(g) depicts the DNN solution for the susceptible population, whereas Fig 4(h) compares the DNN solution with the corresponding fractional-order numerical solution. The two solutions overlap almost completely, demonstrating the excellent approximation capability of the proposed DNN framework and confirming its effectiveness for solving the fractional-order epidemiological model.

**Fig 4:**
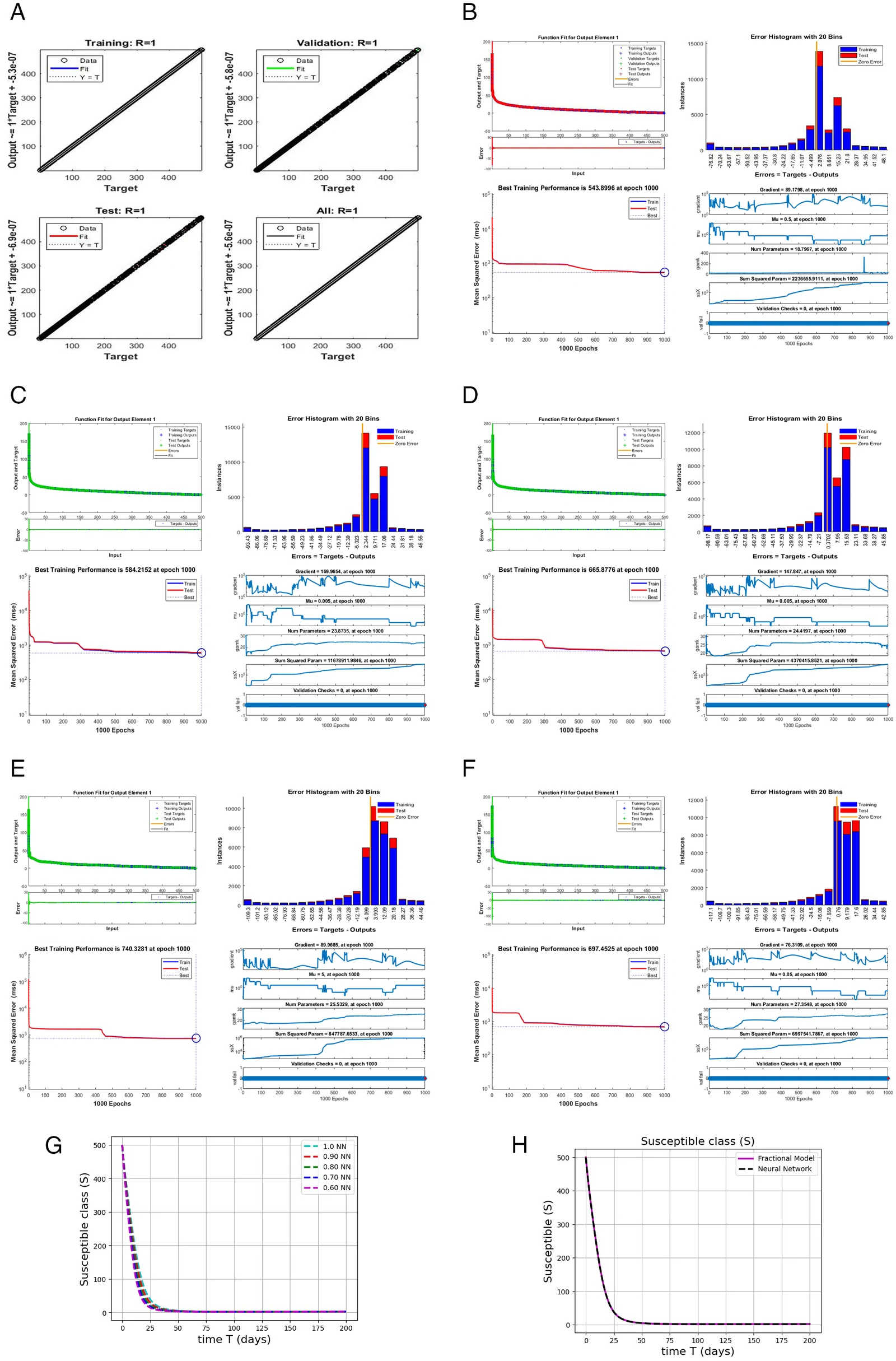
Deep neural network analysis of the dynamics of *S*(*t*): (a) regression performance, (b-f) analysis of the complete datasets corresponding to *S*_*0*_*–S*_*4*_, (g) DNN predictions, and (h) comparison between the fractional-order model and DNN-generated results.

Fig 5 presents the deep neural network (DNN) results for the vaccinated compartment (V) under five different scenarios *V*_0_ to *V*_4_. Fig 5(a) shows the regression analysis for the training, testing, validation, and overall datasets. The regression coefficient is approximately equal to one, indicating an excellent correlation between the network outputs and the target values and confirming the high predictive accuracy of the proposed DNN model.

**Fig 5:**
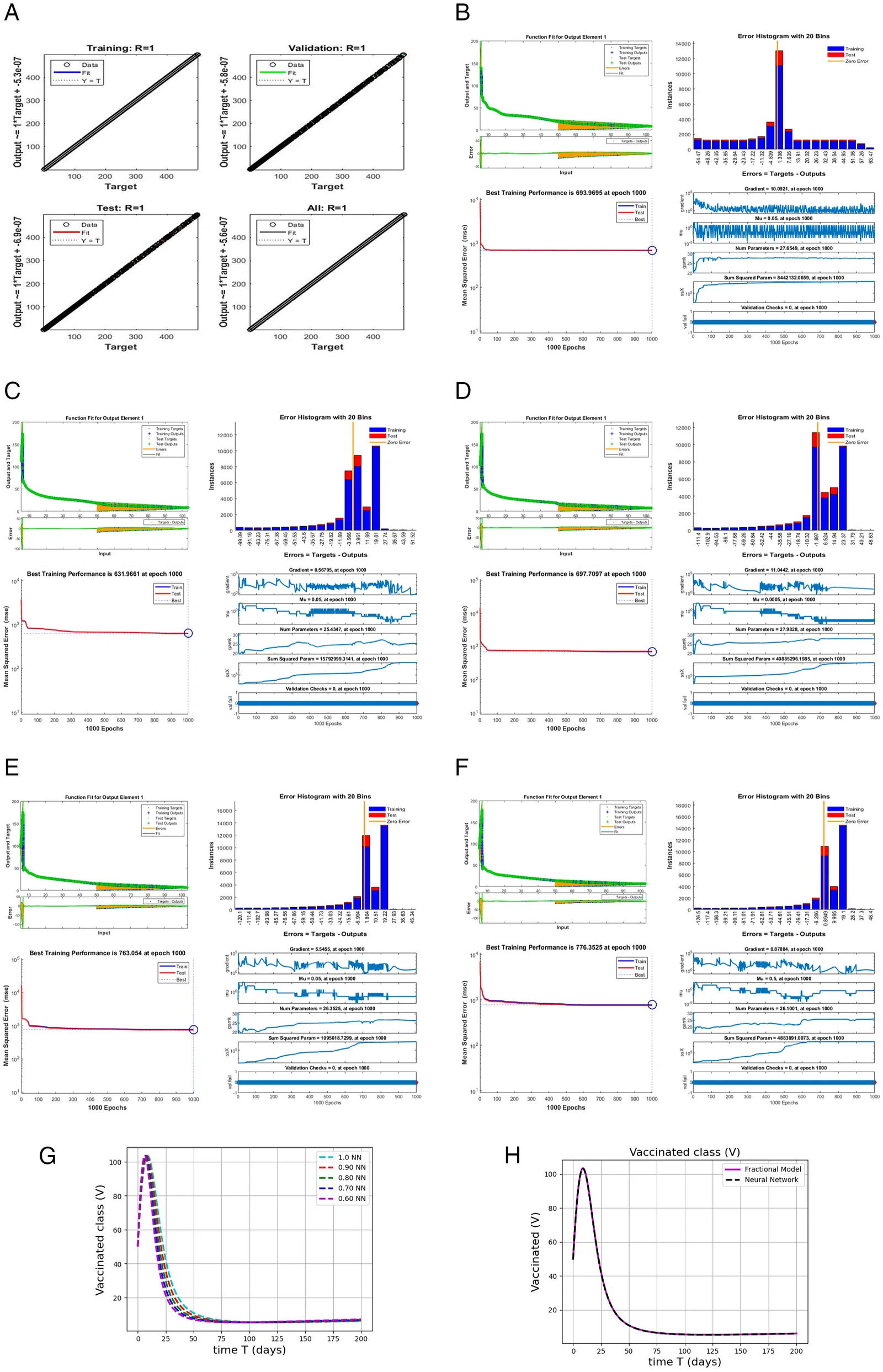
Deep neural network analysis of the dynamics of *V*(*t*): (a) regression performance, (b-f) analysis of the complete datasets corresponding to *V*_0_–*V*_4_, (g) DNN predictions, and (h) comparison between the fractional-order model and DNN-generated results.

Figs 5b–f illustrate the function fitting, error histogram, and training performance for the five scenarios. All networks were trained for 1000 epochs, and the validation checks remained zero throughout the training process, indicating stable convergence without early stopping. The best Mean Squared Error (MSE) values obtained for *V*_0_ to *V*_4_ are 693.9695, 631.9661, 697.7097, 763.054, and 776.3525, respectively. Although the MSE values vary across the scenarios, they remain within an acceptable range, demonstrating the robustness and consistency of the proposed DNN in approximating the fractional-order susceptible dynamics. The corresponding optimization parameters also indicate satisfactory convergence. The gradients for the five cases are 10.0921, 0.56705, 11.0442, 5.5455, and 0.87884, respectively, while the damping parameter μ was automatically adjusted during the training process to improve convergence. The parameter norms and sums of squared parameters remained finite throughout training, further confirming the numerical stability of the network optimization. Finally, Fig 5(g) presents the DNN-based numerical solution for the vaccinated compartment (V), whereas Fig 5(h) compares the DNN approximation with the corresponding fractional-order numerical solution. The close agreement between the two sets of results confirms the robustness, accuracy, and computational efficiency of the proposed DNN framework for solving the model.

Fig 6 presents the deep neural network (DNN) results for the Exposed compartment (E) under five different scenarios *E*_0_ to *E*_4_. Fig 6(a) shows the regression analysis for the training, testing, validation and overall datasets. The regression coefficient is approximately equal to one, indicating an excellent correlation between the network outputs and the target values and confirming the high predictive accuracy of the proposed DNN model. Figs 6b–f present the function fitting and corresponding error histograms (20 bins) for classes *E*_0_ to *E*_4_. The Deep Neural Network (DNN) was trained for 1000 epochs, achieving zero validation checks for all classes. The best training performance (MSE) for classes *E*_0_ to *E*_4_ is 54.5222, 40.5081, 32.6916, 25.0797, and 20.5991, respectively. The corresponding gradient values are 0.085078, 1.8689, 0.25565, 0.075656, and 293.858, whereas the μ values are 0.5, 0.05, 0.0005, 0.5, and 0.5, respectively. Similarly, the number of parameters for classes *E*_0_ to *E*_4_ are 24.7063, 24.1638, 21.1869, 26.4725, and 24.2883, while the sum of squared parameters are 63,231.7276, 75,439.8894, 1,165,412.9074, 2,418,272.0382, and 524,839.3972, respectively. Fig 6(g) presents the neural network (NN) solution for class E, whereas Fig 6(h) illustrates the combined solution obtained from the neural network and the fractional-order model. The close agreement between the NN and fractional solutions demonstrates the high predictive accuracy of the proposed DNN framework for approximating the fractional-order system.

**Fig 6:**
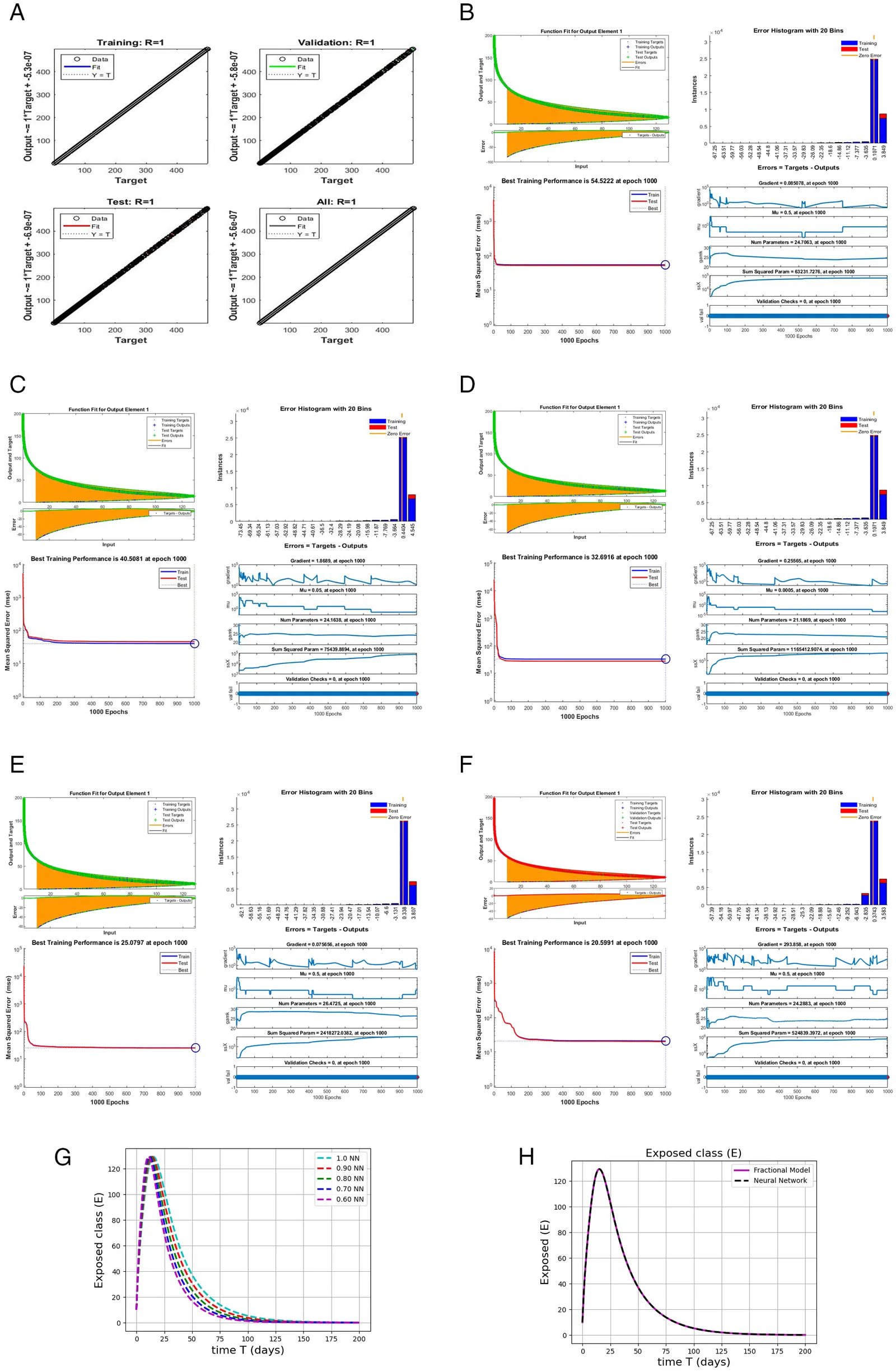
Deep neural network analysis of the dynamics of *E*(*t*): (a) regression performance, (b-f) analysis of the complete datasets corresponding to *E*_0_–*E*_4_, (g) DNN predictions, and (h) comparison between the fractional-order model and DNN-generated results.

Fig 7 presents the deep neural network (DNN) results for the Infected compartment (I) under five different scenarios *I*_0_ to *I*_4_. Fig 7(a) shows the regression analysis for the training, testing, validation, and overall datasets. The regression coefficient is approximately equal to one, indicating an excellent correlation between the network outputs and the target values and confirming the high predictive accuracy of the proposed DNN model. Figs 7(b–f) present the function fitting and corresponding error histograms (20 bins) for classes *I*_0_ to *I*_4_. The Deep Neural Network (DNN) was trained for 1000 epochs, achieving zero validation checks for all classes. The best training performance (MSE) for classes *I*_0_ to *I*_4_ is 45.1371, 33.6922, 27.6821, 24.7474, and 16.2253, respectively. The corresponding gradient values are 3.6186, 7.7478, 25.3805, 1.1853, and 0.24721, whereas the μ values are 5, 0.05, 5, 500, and 0.05, respectively. Similarly, the number of parameters for classes *I*_0_ to *I*_4_ are 20.6352, 25.9894, 20.3949, 16.7357, and 26.6976, while the sum of squared parameters are 34,921.5558, 907,052.7508, 84,848.7575, 37,261.4222, and 3,833,394.4438, respectively. Overall, these results demonstrate that the proposed DNN model achieves consistent convergence with low training errors, stable optimization parameters, and zero validation failures across all classes, confirming its accuracy and robustness in approximating the fractional-order system. Moreover, Fig 7(g) presents the neural network (NN) solution for class I, whereas Fig 7(h) illustrates the combined solution obtained from the neural network and the fractional-order model. The close agreement between the NN and fractional solutions confirms the high predictive accuracy and reliability of the proposed DNN model in approximating the fractional-order system.

**Fig 7:**
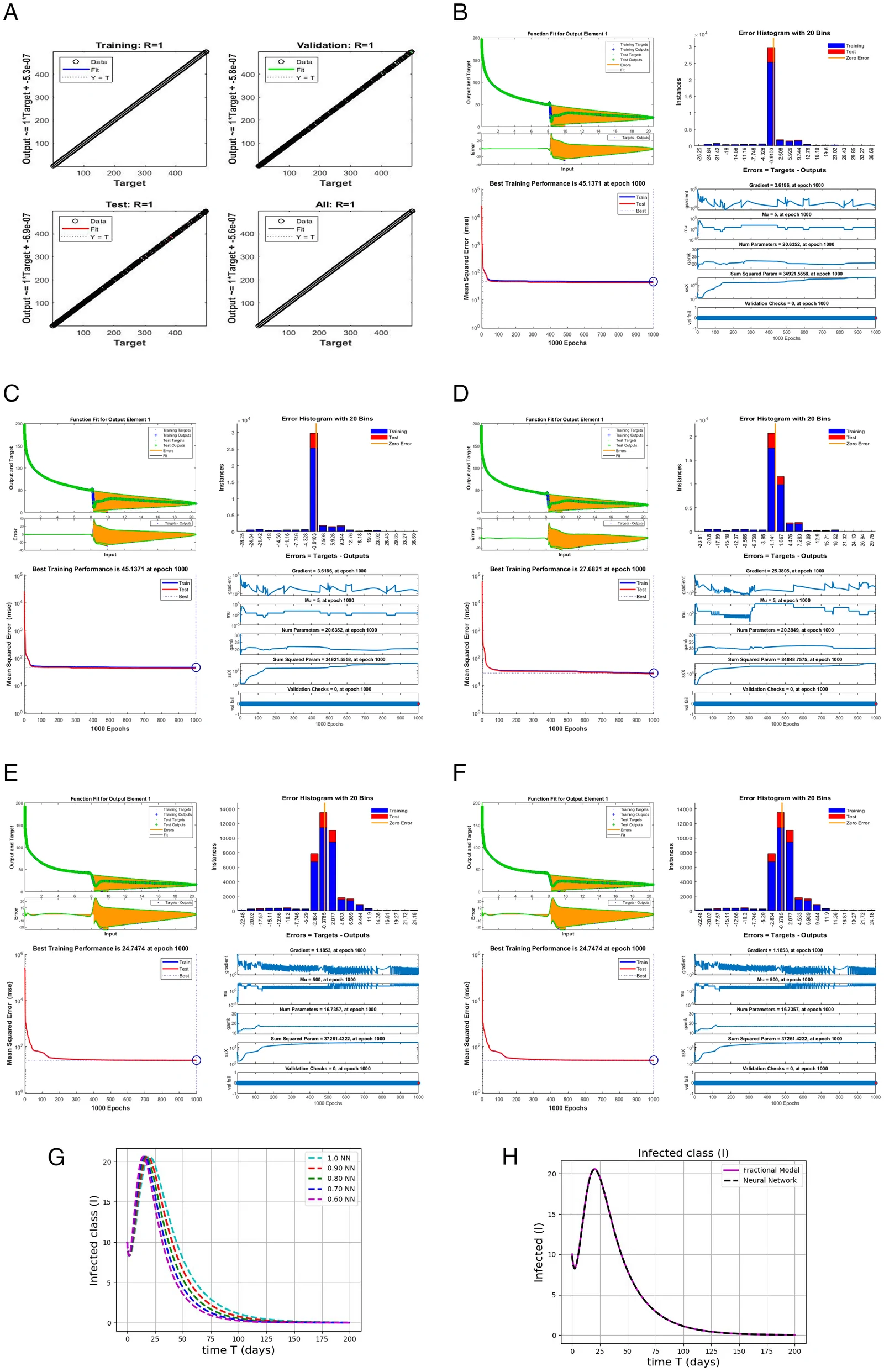
Deep neural network analysis of the dynamics of *I*(*t*): (a) regression performance, (b-f) analysis of the complete datasets corresponding to *I*_0_–*I*_4_, (g) DNN predictions, and (h) comparison between the fractional-order model and DNN-generated results.

Fig 8(a) presents the regression analysis for the recovered compartment (R) along with the corresponding output obtained using the Deep Neural Network (DNN). The regression coefficient is 1.0, indicating an excellent agreement between the predicted and target values.

**Fig 8:**
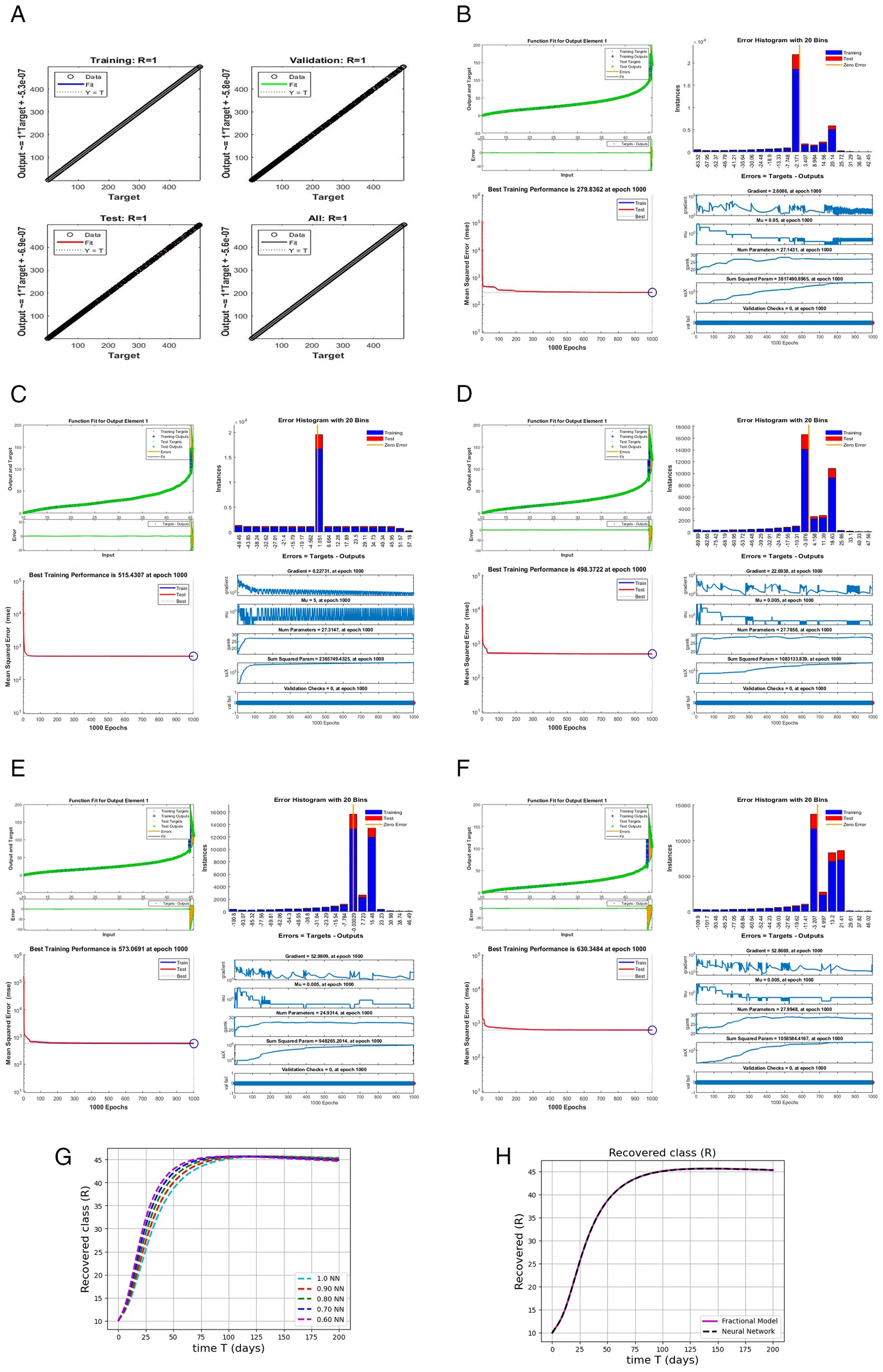
Deep neural network analysis of the dynamics of *R*(*t*): (a) regression performance, (b-f) analysis of the complete datasets corresponding to *R*_0_–*R*_4_, (g) DNN predictions, and (h) comparison between the fractional-order model and DNN-generated results.

Figs 8b–f present the function fitting and corresponding error histograms (20 bins) for classes $\left(R_{0}-R_{4}\right)$, respectively. The Deep Neural Network (DNN) was trained for 1000 epochs, achieving zero validation checks for all classes. The best training performance (MSE) for classes $\left(R_{0}-R_{4}\right)$ is 279.8362, 515.4307, 498.3722, 630.3484, and 573.0691, respectively. The corresponding gradient values are 2.3541, 0.22731, 22.6938, 52.8669, and 52.9809, while the μ values are 0.005, 5, 0.005, 0.005, and 0.05, respectively. Similarly, the number of parameters for classes $\left(R_{0}-R_{4}\right)$ are 29.9783, 27.3147, 27.7856, 27.9948, and 24.9314, whereas the corresponding sum of squared parameters are 2,611,366.3197, 2,365,749.4325, 1,083,133.8390, 1,058,584.4167, and 948,265.2014, respectively. Overall, the DNN exhibited stable convergence with satisfactory training performance and zero validation failures across all subclasses, demonstrating its effectiveness and robustness in approximating the solution of class R. Fig 8(g) presents the neural network (NN) solution for class R, demonstrating the predicted response generated by the trained DNN model. Fig 8(h) shows the combined solution obtained by integrating the neural network (NN) predictions with the fractional-order numerical solution. The close agreement between the two solutions confirms the effectiveness, robustness, and accuracy of the proposed DNN approach in approximating the fractional model for class R.

## 8. Conclusion

Mathematical modeling of Norovirus outbreaks has given a useful understanding of the mechanisms of transmission and control. In this paper, a novel fractional-order mathematical model based on the Caputo–Fabrizio operator is developed to analyze the transmission dynamics of norovirus, a highly contagious disease that causes severe outbreaks of gastroenteritis worldwide. The nonlocal and memory-dependent nature of the disease transmission processes, which are often neglected by the integer order models, is well represented by the proposed system because of the integration of the aspect of a state known as fractional calculus is incorporated in the modeling framework. The analytical analysis established the existence and uniqueness of the model solution using the fixed-point theorem, ensuring that the system is mathematically well-posed. The stability analysis further showed that the disease-free equilibrium is more stable at lower fractional orders, indicating that memory effects can reduce the rate of disease transmission. Furthermore, the numerical simulations are carried out by using the three-step Adams–Bashforth scheme, demonstrating consistency between the model predictions and the observed epidemiological trends. Moreover, a deep neural network (DNN) is employed to solve the model, and its performance is evaluated by comparing its predictions with those obtained using a classical numerical method. The findings demonstrate that fractional-order dynamics effectively capture the persistence and heterogeneity of norovirus transmission. In particular, increased vaccination coverage and treatment efficacy significantly reduce infection and improve disease control. These results can support public health authorities in developing effective strategies for preventing norovirus outbreaks, particularly in high-risk settings such as schools, hospitals, and care homes. Overall, the Caputo–Fabrizio framework provides a useful tool for incorporating memory and nonlocal effects into epidemic modeling. Future work may extend the model by incorporating spatial dynamics, stochastic effects, and machine-learning-based parameter estimation to enhance its predictive capability and practical relevance.

## Supporting information

S1 File Data. (ZIP)
