# Supplementary material for: Deep learning-based predictions and analysis of Norovirus dynamics using a fractional-order mathematical model
Source: PLoS One. 2026 Oct 14;21(10):e0359983. doi: 10.1371/journal.pone.0359983 (PMC13667835; doi:10.1371/journal.pone.0359983)
Supplement: Supplementary file 2 [file pone.0359983.s004.pdf]

Overall Decision: Major Revision Required

The manuscript attempts to combine fractional calculus (Caputo-Fabrizio), a compartmental model (SVEIR), numerical simulations (Adams-Bashforth), and Deep Neural Networks (DNN). While the topic is timely, the current execution suffers from lack of biological justification, mathematical inconsistencies, poor presentation of DNN results, and missing critical information (units, parameter sources, compartment diagram). The claimed "memory effect" of fractional order is not mechanistically linked to norovirus biology.

1) In introduction, the *Motivation for fractional-order is weak and generic.* The authors state fractional calculus captures "memory and nonlocal processes" but never explain what specific memory in norovirus transmission justifies a fractional derivative.
2) Norovirus infection lasts approx. 2 to 3 days in healthy individuals. What biological mechanism requires a memory kernel spanning weeks or months?
3) The statement *"classical integer-order models characterize memory only to a limited extent"* is vague. No example or citation is given where integer-order SEIR-type models failed for norovirus.
4) Caputo Fabrizio (CF) derivative uses an exponential kernel (no singularity), while Caputo has a power-law kernel. For disease with short infectious period, the biological rationale for CF over standard Caputo is not provided. The authors mention "Atangana–Baleanu fractional derivative" but then use CF. This is confusing and not justified.
5) The abstract and introduction repeat the same sentences almost verbatim (e.g., "extremely contagious and has a significant impact on the gastrointestinal tract").
6) The statement "Deep Neural Networks provide a flexible framework without discretization" is true in principle, but later the DNN is *trained on data from Adams-Bashforth*. That defeats the purpose — it's not a truly independent solver.
7) No mention of prior fractional models for norovirus (if any) to establish novelty.
8) In Model formulation, schematic diagram for the proposed model is missing.
9) In table, Parameter values are given without sources or units.
10) Vaccine efficacy is very high. Is that realistic for norovirus vaccines still in development? Citation needed.
11) What is the use of theoretical theorems in this section? Theorems on existence, uniqueness, and Ulam-Hyers stability (Section 3, page 76 onward) are mathematically standard for fractional systems but are not connected to norovirus biology. They do not inform parameter estimation, control strategies, or predictions.
12) In section 4 and 5, *The DNN is NOT solving the fractional model directly. Is that correct?* The authors write: *"The datasets, obtained from the piecewise Adams-Bashforth numerical methods, were used to train the DNN"* (page 90). This means the DNN is merely *fitting* the numerical solution, not approximating the fractional differential equation independently.
13) Equation (67) defines a residual loss, but the authors then admit they used Adams-Bashforth data. This seems a contradiction.

14) The description of results is vague in terms of MSE and epochs. Also make sure all figures are explained well in the text.
