# Supplementary material for: Deep learning-based predictions and analysis of Norovirus dynamics using a fractional-order mathematical model
Source: PLoS One. 2026 Oct 14;21(10):e0359983. doi: 10.1371/journal.pone.0359983 (PMC13667835; doi:10.1371/journal.pone.0359983)
Supplement: Supplementary file 3 [file pone.0359983.s002.pdf]

Response to Reviewers

Journal Requirements:

When submitting your revision, we need you to address these additional requirements.

1. Please ensure that your manuscript meets PLOS ONE's style requirements, including those for file naming. The PLOS ONE style templates can be found at

   https://journals.plos.org/plosone/s/file?id=wjVg/PLOSOne_formatting_sample_main_body.pdf and

   https://journals.plos.org/plosone/s/file?id=ba62/PLOSOne_formatting_sample_title_authors_affiliations.pdf

Author Response: Formatted according to journal guide lines

2. Please note that PLOS One has specific guidelines on code sharing for submissions in which author-generated code underpins the findings in the manuscript. In these cases, we expect all author-generated code to be made available without restrictions upon publication of the work. Please review our guidelines at https://journals.plos.org/plosone/s/materials-and-software-sharing#loc-sharing-code and ensure that your code is shared in a way that follows best practice and facilitates reproducibility and reuse.

   Author Response: We confirm that all authors will adhere to the PLOS ONE guidelines on code sharing. All author-generated code used in this study will be made publicly available without restrictions in accordance with these guidelines.

3. In the online submission form, you indicated that "All relevant data from this study will be made available upon request."

   All PLOS journals now require all data underlying the findings described in their manuscript to be freely available to other researchers, either 1. In a public repository, 2. Within the manuscript itself, or 3. Uploaded as supplementary information.

   This policy applies to all data except where public deposition would breach compliance with the protocol approved by your research ethics board. If your data cannot be made publicly available for ethical or legal reasons (e.g., public availability would compromise patient privacy), please explain your reasons on resubmission and your exemption request will be escalated for approval.

   Author Response: We confirm that all data underlying the findings of this study will be made fully and freely available in accordance with PLOS ONE data sharing policies. The data will be provided either within the manuscript, as supplementary information, or through a publicly accessible repository.

4. Please ensure that you refer to Figure 5 and 7 in your text as, if accepted, production will need this reference to link the reader to the figure.

   Author Response: We have revised the manuscript to ensure that Figures 5 and 7 are appropriately referenced in the main text. The corresponding descriptions have been added to guide the reader and ensure proper linkage to these figures.

5. Please include captions for your Supporting Information files at the end of your manuscript, and update any in-text citations to match accordingly. Please see our Supporting Information guidelines for more information: http://journals.plos.org/plosone/s/supporting-information.

   Author Response: We have revised the manuscript to include captions for all Supporting Information files in accordance with PLOS ONE guidelines.

6. If the reviewer comments include a recommendation to cite specific previously published works, please review and evaluate these publications to determine whether they are relevant and should be cited. There is no requirement to cite these works unless the editor has indicated otherwise.

Author Response: We will carefully review the publications suggested by the reviewer and incorporate only those references that are relevant to our study into the revised manuscript. References that are not directly related to the scope of our work will not be included.

Additional Editor Comments:

Please revise the manuscript and confirm the PDF before submission to the journal. All the figures are missing.

Author Response: updated in revised version
