# Supplementary material for: Deep learning-based predictions and analysis of Norovirus dynamics using a fractional-order mathematical model
Source: PLoS One. 2026 Oct 14;21(10):e0359983. doi: 10.1371/journal.pone.0359983 (PMC13667835; doi:10.1371/journal.pone.0359983)
Supplement: Supplementary file 4 [file pone.0359983.s005.pdf]

Review Report for PLOS ONE (PONE-D-26-17282R1)

Overall Decision: Major Revision Required

The manuscript attempts to combine fractional calculus (Caputo-Fabrizio), a compartmental model (SVEIR), numerical simulations (Adams-Bashforth), and Deep Neural Networks (DNN). While the topic is timely, the current execution suffers from lack of biological justification, mathematical inconsistencies, poor presentation of DNN results, and missing critical information (units, parameter sources, compartment diagram). The claimed "memory effect" of fractional order is not mechanistically linked to norovirus biology.

1) In introduction, the *Motivation for fractional-order is weak and generic.* The authors state fractional calculus captures "memory and nonlocal processes" but never explain what specific memory in norovirus transmission justifies a fractional derivative.

   Author Response: Introduction has been updated with proper citations and highlighted in the revised manuscript file.

2) Norovirus infection lasts approx. 2 to 3 days in healthy individuals. What biological mechanism requires a memory kernel spanning weeks or months?

   Author Response: This is the insightful observation from reviewer's. We agree that the norovirus infection lasts approximately 2 to 3 days in healthy individuals. Basically, norovirous transmission dynamics involve memory related effects due to environmental viral persistence, asymptomatic transmission and influence of previous infections on future spread. Fractional order models capture these historical dependencies and and provide a more realistic description of novovirous transmission. Reference [23]

3) The statement *"classical integer-order models characterize memory only to a limited extent"* is vague. No example or citation is given where integer-order SEIR-type models failed for norovirus.

   Author Response: We agree that the previous statement was too general. We have revised the manuscript to clarify that classical integer-order SEIR-type models are useful for describing norovirus transmission but assume that the current state depends only on instantaneous conditions and may not explicitly incorporate history-dependent effects such as environmental persistence, delayed transmission, and temporary immunity. Appropriate references have been added to support this clarification. Reference [24]

4) Caputo Fabrizio (CF) derivative uses an exponential kernel (no singularity), while Caputo has a power-law kernel. For disease with short infectious period, the biological rationale for CF over standard Caputo is not provided. The authors mention "Atangana–Baleanu fractional derivative" but then use CF. This is confusing and not justified.

   Author Response: We know that Caputo, Caputo-Fabrizio and Atangana- Baleanu are different operators are distinct fractional operators characterized by different kernel functions and properties. The confusing statement has been removed from revised manuscript file.

5) The abstract and introduction repeat the same sentences almost verbatim (e.g., "extremely contagious and has a significant impact on the gastrointestinal tract").
Author Response: Removed the repeated sentences and updated in the manuscript file

6) The statement "Deep Neural Networks provide a flexible framework without discretization" is true in principle, but later the DNN is *trained on data from Adams-Bashforth*. That defeats the purpose — it's not a truly independent solver.

Author Response: The proposed DNN is trained directly using the differential equations along with conditions. No discretized numerical solution is used during the training process. The Adams-Bashforth method is employed solely to validate the accuracy of the proposed DNN by comparing the resulting numerical solutions.

7) No mention of prior fractional models for norovirus (if any) to establish novelty.

Author Response: The present work is primarily motivated by the studies reported in [19, 22, 23], which emphasize the importance of fractional-order norovirus models and reveal the need for accurate and efficient computational approaches. Moreover, we develop a Deep Neural Network (DNN)-based framework to obtain reliable numerical solutions for the proposed model.

8) In Model formulation, schematic diagram for the proposed model is missing.
Author Response: The schematic diagram added in the revised file

9) In table, Parameter values are given without sources or units.
Author Response: Updated with unit and source

10) Vaccine efficacy is very high. Is that realistic for norovirus vaccines still in development? Citation needed.

Author Response: Yes, norovirus vaccines still in development. The vaccine efficacy parameter used in this study represents a hypothetical value adopted for mathematical modeling and development analysis. It is intended to investigate the potential impact of a highly effective vaccine on disease transmission rather than to reflect the efficacy of an approved vaccine. This assumption is consistnent with modeling studies that evaluate prospective vaccination strategies and is supported by the current literature on norovirus vaccine development.

We updated the introduction part with the following latest references related to vaccines development [10,11].

[10] Tan, Ming. "The Impact of Norovirus on Children and Adolescents: Implications for Ongoing Vaccine Development." *Current Pediatrics Reports* 13.1 (2025): 21.

[11] Lindesmith, Lisa C., et al. "Norovirus GII. 4 serological repertoire in older adults after GII. 4 ancestral variant vaccination: a post-hoc analysis of a randomised, double-blind, phase 2 trial." *The Lancet Microbe* 7.6 (2026).

11) What is the use of theoretical theorems in this section? Theorems on existence, uniqueness, and Ulam-Hyers stability (Section 3, page 76 onward) are mathematically standard for fractional systems but are not connected to norovirus biology. They do not inform parameter estimation, control strategies, or predictions.

Author Response: we agree that the existence, uniqueness, and Ulam-Hyers stability theorems are standard mathematical results for fractional differential systems. However, their inclusion is essential to establish the mathematical well posedness of the proposed norovirus transmission model before performing numerical simulation. Specifically, the existence and uniqueness results guarantee that the model admits a well defined and physically meaningful solution, while the Ulam-Hyers stability analysis demonstrates that the solution remains stable under small perturbations in the initial condions or model parameters. These theoretical properties ensure that the predicted disease dynamics are mathematically reliable and that the subsequent numerical and DNN based results are based on a strong mathematical foundation.

12) In section 4 and 5, *The DNN is NOT solving the fractional model directly. Is that correct?* The authors write: *"The datasets, obtained from the piecewise Adams-Bashforth numerical methods, were used to train the DNN"* (page 90). This means the DNN is merely *fitting* the numerical solution, not approximating the fractional differential equation independently.

Author Response: The DNN solves the fractional model directly, as stated by the authors on the same page (90) "the network learn purely from the ODEs structure and initial conditions". Therefore, the sentence highlighted by the reviewer, *"The datasets, obtained from the piecewise Adams-Bashforth numerical methods, were used to train the DNN",* is indeed contradictory. In response to the reviewer comment, we have removed this sentence from the revised manuscript to eliminate the inconsistency.

13) Equation (67) defines a residual loss, but the authors then admit they used Adams-Bashforth data. This seems a contradiction.

Author Response: This contradiction has already been addressed in response to the previous comment and has been corrected in the revised manuscript.

14) Also make sure all figures are explained well in the text.

Author Response: All Figures have been carefully reviewed, appropriately described and properly referenced in the text. The descriptions of results in terms of MSE and number of training epochs have been thoroughly checked and updated throughout the revised manuscript.
