# Supplementary material for: Deep learning-based predictions and analysis of Norovirus dynamics using a fractional-order mathematical model
Source: PLoS One. 2026 Oct 14;21(10):e0359983. doi: 10.1371/journal.pone.0359983 (PMC13667835; doi:10.1371/journal.pone.0359983)
Supplement: Supplementary file 5 [file pone.0359983.s006.pdf]

Response to Reviewers

The authors need to addressed the following comments

1 language improvement

Authors Response: We have carefully revised the manuscript to improve the overall language, grammar, sentence structure, and readability.

2 consistency of mathematical notation

Authors Response: All relevant mathematical symbols, variables, parameters, and equations have been checked and standardized accordingly.

3 Improvement of figure captions

Authors Response: We have revised and improved the captions of all figures to make them more descriptive, clear, and self-explanatory.

4 Strengthening of the conclusion.

We have strengthened the conclusion section by providing a clearer summary of the main findings, highlighting the significance of the proposed approach, and discussing the potential implications and future directions of the study.
