# Supplementary material for: Deep learning-based predictions and analysis of Norovirus dynamics using a fractional-order mathematical model
Source: PLoS One. 2026 Oct 14;21(10):e0359983. doi: 10.1371/journal.pone.0359983 (PMC13667835; doi:10.1371/journal.pone.0359983)
Supplement: Supplementary file 7 [file pone.0359983.s003.docx]

Review for PLOS ONE (PONE-D-26-17282R1)

Deep Learning-Based Predictions and Analysis of Norovirus Dynamics using a Fractional-order Mathematical Model

This manuscript proposes a fractional-order SVEIR model for norovirus transmission using the Caputo-Fabrizio derivative. The authors present fixed-point existence/uniqueness proofs, numerical simulations using a three-step Adams-Bashforth scheme, and a deep neural network (DNN) approximation with a novel activation function (NGNDG-AF). The manuscript addresses an interesting application of fractional calculus to norovirus modeling, and the existence/uniqueness analysis is a positive contribution. However, the current version contains technical errors. These issues must be addressed before the publication.

I recommend Minor Revision and encourage the authors to carefully address each of the points below.

1. The manuscript contains numerous grammatical errors and awkward phrasings make it correct

2. The abstract is overly long and repetitive. Many sentences could be condensed.

3. The fractional-order model is presented in integer-order form (Equation 1), but the analysis and numerical methods assume a Caputo-Fabrizio formulation. The authors never explicitly write the fractional version of the system.

4. In Section 4 (Main Results), the integral representation uses a parameter `η` that is never clearly defined in relation to the fractional order `ζ`. This creates confusion throughout the existence/uniqueness proofs.

5. The model includes disease-induced mortality `d = 0.33`, which is extremely high (33% of infected individuals die). Norovirus mortality is typically well below 1% in developed countries. This parameter likely represents something else or is grossly miscalibrated.

6. Figure captions are missing thoroughly.
