# Supplementary material for: Deep learning-based predictions and analysis of Norovirus dynamics using a fractional-order mathematical model
Source: PLoS One. 2026 Oct 14;21(10):e0359983. doi: 10.1371/journal.pone.0359983 (PMC13667835; doi:10.1371/journal.pone.0359983)
Supplement: Peer review history

# Editor report: Decision Letter 0

**DOI:** 10.1371/journal.pone.0359983.r001

**Authors:** Asad Ullah

11 Apr 2026

Dear Dr.  Ali,

Thank you for submitting your manuscript to PLOS ONE. After careful consideration, we feel that it has merit but does not fully meet PLOS ONE’s publication criteria as it currently stands. Therefore, we invite you to submit a revised version of the manuscript that addresses the points raised by the editor.

==============================

**Authors are needs to submit a revised version of the manuscript by confirming their PDF before submission to the journal. In the current form, all the figures are missing.**

==============================

Please submit your revised manuscript by  May 26 2026 11:59PM. If you will need more time than this to complete your revisions, please reply to this message or contact the journal office at plosone@plos.org. When you're ready to submit your revision, log on to https://www.editorialmanager.com/pone/ and select the 'Submissions Needing Revision' folder to locate your manuscript file.

- A letter that responds to each point raised by the academic editor and reviewer(s). You should upload this letter as a separate file labeled 'Response to Reviewers'.
- A marked-up copy of your manuscript that highlights changes made to the original version. You should upload this as a separate file labeled 'Revised Manuscript with Track Changes'.
- An unmarked version of your revised paper without tracked changes. You should upload this as a separate file labeled 'Manuscript'.

If you would like to make changes to your financial disclosure, please include your updated statement in your cover letter. Guidelines for resubmitting your figure files are available below the reviewer comments at the end of this letter.

If applicable, we recommend that you deposit your laboratory protocols in protocols.io to enhance the reproducibility of your results. Protocols.io assigns your protocol its own identifier (DOI) so that it can be cited independently in the future. For instructions see: https://journals.plos.org/plosone/s/submission-guidelines#loc-laboratory-protocols. Additionally, PLOS ONE offers an option for publishing peer-reviewed Lab Protocol articles, which describe protocols hosted on protocols.io. Read more information on sharing protocols at https://plos.org/protocols?utm_medium=editorial-email&utm_source=authorletters&utm_campaign=protocols.

As the corresponding author, your ORCID iD is verified in the submission system and will appear in the published article. PLOS supports the use of ORCID, and we encourage all coauthors to register for an ORCID iD and use it as well. Please encourage your coauthors to verify their ORCID iD within the submission system before final acceptance, as unverified ORCID iDs will not appear in the published article. *Only*  the individual author can complete the verification step; PLOS staff *cannot*  verify ORCID iDs on behalf of authors.

We look forward to receiving your revised manuscript.

Kind regards,

Asad Ullah, Postdoc

Academic Editor

PLOS One

Journal Requirements:

When submitting your revision, we need you to address these additional requirements.

1.Please ensure that your manuscript meets PLOS ONE's style requirements, including those for file naming. The PLOS ONE style templates can be found at

https://journals.plos.org/plosone/s/file?id=wjVg/PLOSOne_formatting_sample_main_body.pdf and

https://journals.plos.org/plosone/s/file?id=ba62/PLOSOne_formatting_sample_title_authors_affiliations.pdf

2. Please note that PLOS One has specific guidelines on code sharing for submissions in which author-generated code underpins the findings in the manuscript. In these cases, we expect all author-generated code to be made available without restrictions upon publication of the work. Please review our guidelines at https://journals.plos.org/plosone/s/materials-and-software-sharing#loc-sharing-code and ensure that your code is shared in a way that follows best practice and facilitates reproducibility and reuse.

3. In the online submission form, you indicated that “All relevant data from this study will be made available upon request.”

All PLOS journals now require all data underlying the findings described in their manuscript to be freely available to other researchers, either 1. In a public repository, 2. Within the manuscript itself, or 3. Uploaded as supplementary information.

This policy applies to all data except where public deposition would breach compliance with the protocol approved by your research ethics board. If your data cannot be made publicly available for ethical or legal reasons (e.g., public availability would compromise patient privacy), please explain your reasons on resubmission and your exemption request will be escalated for approval.

4. Please ensure that you refer to Figure 5 and 7 in your text as, if accepted, production will need this reference to link the reader to the figure.

5. Please include captions for your Supporting Information files at the end of your manuscript, and update any in-text citations to match accordingly. Please see our Supporting Information guidelines for more information: http://journals.plos.org/plosone/s/supporting-information.

6. If the reviewer comments include a recommendation to cite specific previously published works, please review and evaluate these publications to determine whether they are relevant and should be cited. There is no requirement to cite these works unless the editor has indicated otherwise.

Additional Editor Comments:

Please revise the manuscript and confirm the PDF before submission to the journal. All the figures are missing.

[Note: HTML markup is below. Please do not edit.]

[NOTE: If reviewer comments were submitted as an attachment file, they will be attached to this email and accessible via the submission site. Please log into your account, locate the manuscript record, and check for the action link "View Attachments". If this link does not appear, there are no attachment files.]

To ensure your figures meet our technical requirements, please review our figure guidelines: https://journals.plos.org/plosone/s/figures

You may also use PLOS’s free figure tool, NAAS, to help you prepare publication quality figures: https://journals.plos.org/plosone/s/figures#loc-tools-for-figure-preparation.

NAAS will assess whether your figures meet our technical requirements by comparing each figure against our figure specifications.

# Author comment: Author response to Decision Letter 1

**DOI:** 10.1371/journal.pone.0359983.r002

14 May 2026

Response to Editor/Reviewers

Journal Requirements:

When submitting your revision, we need you to address these additional requirements.

1. Please ensure that your manuscript meets PLOS ONE's style requirements, including those for file naming. The PLOS ONE style templates can be found at

https://journals.plos.org/plosone/s/file?id=wjVg/PLOSOne_formatting_sample_main_body.pdf and

https://journals.plos.org/plosone/s/file?id=ba62/PLOSOne_formatting_sample_title_authors_affiliations.pdf

Author Response: Formatted according to journal guide lines

2. Please note that PLOS One has specific guidelines on code sharing for submissions in which author-generated code underpins the findings in the manuscript. In these cases, we expect all author-generated code to be made available without restrictions upon publication of the work. Please review our guidelines at https://journals.plos.org/plosone/s/materials-and-software-sharing#loc-sharing-code and ensure that your code is shared in a way that follows best practice and facilitates reproducibility and reuse.

Author Response: We confirm that all authors will adhere to the PLOS ONE guidelines on code sharing. All author-generated code used in this study will be made publicly available without restrictions in accordance with these guidelines.

3. In the online submission form, you indicated that “All relevant data from this study will be made available upon request.”

All PLOS journals now require all data underlying the findings described in their manuscript to be freely available to other researchers, either 1. In a public repository, 2. Within the manuscript itself, or 3. Uploaded as supplementary information.

This policy applies to all data except where public deposition would breach compliance with the protocol approved by your research ethics board. If your data cannot be made publicly available for ethical or legal reasons (e.g., public availability would compromise patient privacy), please explain your reasons on resubmission and your exemption request will be escalated for approval.

Author Response: We confirm that all data underlying the findings of this study will be made fully and freely available in accordance with PLOS ONE data sharing policies. The data will be provided either within the manuscript, as supplementary information, or through a publicly accessible repository.

4. Please ensure that you refer to Figure 5 and 7 in your text as, if accepted, production will need this reference to link the reader to the figure.

Author Response: We have revised the manuscript to ensure that Figures 5 and 7 are appropriately referenced in the main text. The corresponding descriptions have been added to guide the reader and ensure proper linkage to these figures.

5. Please include captions for your Supporting Information files at the end of your manuscript, and update any in-text citations to match accordingly. Please see our Supporting Information guidelines for more information: http://journals.plos.org/plosone/s/supporting-information.

Author Response: We have revised the manuscript to include captions for all Supporting Information files in accordance with PLOS ONE guidelines.

6. If the reviewer comments include a recommendation to cite specific previously published works, please review and evaluate these publications to determine whether they are relevant and should be cited. There is no requirement to cite these works unless the editor has indicated otherwise.

Author Response: We will carefully review the publications suggested by the reviewer and incorporate only those references that are relevant to our study into the revised manuscript. References that are not directly related to the scope of our work will not be included.

Additional Editor Comments:

Please revise the manuscript and confirm the PDF before submission to the journal. All the figures are missing.

Author Response: updated in revised version

Attachment Submitted filename: Response to Reviewers.pdf

# Peer review: Decision Letter 1

**DOI:** 10.1371/journal.pone.0359983.r003

**Authors:** Asad Ullah

11 Jun 2026

Dear Dr. Ali,

Academic Editor Comments:

The article needs to follow the standard PLOS ONE format in all aspect including references and figures style. All figures need o be in good resolution. The language of the article needs strong editing before re-submission.

Please submit your revised manuscript by Jul 26 2026 11:59PM. If you will need more time than this to complete your revisions, please reply to this message or contact the journal office at plosone@plos.org. When you're ready to submit your revision, log on to https://www.editorialmanager.com/pone/ and select the 'Submissions Needing Revision' folder to locate your manuscript file.

- A letter that responds to each point raised by the academic editor and reviewer(s). You should upload this letter as a separate file labeled 'Response to Reviewers'.
- A marked-up copy of your manuscript that highlights changes made to the original version. You should upload this as a separate file labeled 'Revised Manuscript with Track Changes'.
- An unmarked version of your revised paper without tracked changes. You should upload this as a separate file labeled 'Manuscript'.

If you would like to make changes to your financial disclosure, please include your updated statement in your cover letter. Guidelines for resubmitting your figure files are available below the reviewer comments at the end of this letter.

If applicable, we recommend that you deposit your laboratory protocols in protocols.io to enhance the reproducibility of your results. Protocols.io assigns your protocol its own identifier (DOI) so that it can be cited independently in the future. For instructions see: https://journals.plos.org/plosone/s/submission-guidelines#loc-laboratory-protocols. Additionally, PLOS ONE offers an option for publishing peer-reviewed Lab Protocol articles, which describe protocols hosted on protocols.io. Read more information on sharing protocols at https://plos.org/protocols?utm_medium=editorial-email&utm_source=authorletters&utm_campaign=protocols.

As the corresponding author, your ORCID iD is verified in the submission system and will appear in the published article. PLOS supports the use of ORCID, and we encourage all coauthors to register for an ORCID iD and use it as well. Please encourage your coauthors to verify their ORCID iD within the submission system before final acceptance, as unverified ORCID iDs will not appear in the published article. *Only*  the individual author can complete the verification step; PLOS staff *cannot*  verify ORCID iDs on behalf of authors.

We look forward to receiving your revised manuscript.

Kind regards,

Asad Ullah, Postdoc

Academic Editor

PLOS One

Journal Requirements:

If the reviewer comments include a recommendation to cite specific previously published works, please review and evaluate these publications to determine whether they are relevant and should be cited. There is no requirement to cite these works unless the editor has indicated otherwise.

[Note: HTML markup is below. Please do not edit.]

Reviewers' comments:

Reviewer's Responses to Questions

**Comments to the Author**

Reviewer #1: All comments have been addressed

Reviewer #2: All comments have been addressed

**********

2. Is the manuscript technically sound, and do the data support the conclusions?

Reviewer #1: Yes

Reviewer #2: Yes

**********

3. Has the statistical analysis been performed appropriately and rigorously?

Reviewer #1: Yes

Reviewer #2: Yes

**********

4. Have the authors made all data underlying the findings in their manuscript fully available?

The PLOS Data policy

Reviewer #1: Yes

Reviewer #2: Yes

**********

5. Is the manuscript presented in an intelligible fashion and written in standard English?

Reviewer #1: Yes

Reviewer #2: Yes

**********

Reviewer #1: Review for PLOS ONE (PONE-D-26-17282R1)

Deep Learning-Based Predictions and Analysis of Norovirus Dynamics using a Fractional-order Mathematical Model

This manuscript proposes a fractional-order SVEIR model for norovirus transmission using the Caputo-Fabrizio derivative. The authors present fixed-point existence/uniqueness proofs, numerical simulations using a three-step Adams-Bashforth scheme, and a deep neural network (DNN) approximation with a novel activation function (NGNDG-AF). The manuscript addresses an interesting application of fractional calculus to norovirus modeling, and the existence/uniqueness analysis is a positive contribution. However, the current version contains technical errors. These issues must be addressed before the publication.

I recommend Minor Revision and encourage the authors to carefully address each of the points below.

1. The manuscript contains numerous grammatical errors and awkward phrasings make it correct

2. The abstract is overly long and repetitive. Many sentences could be condensed.

3. The fractional-order model is presented in integer-order form (Equation 1), but the analysis and numerical methods assume a Caputo-Fabrizio formulation. The authors never explicitly write the fractional version of the system.

4. In Section 4 (Main Results), the integral representation uses a parameter `η` that is never clearly defined in relation to the fractional order `ζ`. This creates confusion throughout the existence/uniqueness proofs.

5. The model includes disease-induced mortality `d = 0.33`, which is extremely high (33% of infected individuals die). Norovirus mortality is typically well below 1% in developed countries. This parameter likely represents something else or is grossly miscalibrated.

6. Figure captions are missing thoroughly.

Reviewer #2: (No Response)

**********

what does this mean?). If published, this will include your full peer review and any attached files.

If you choose “no”, your identity will remain anonymous but your review may still be made public.

**Do you want your identity to be public for this peer review?**  For information about this choice, including consent withdrawal, please see our Privacy Policy

Reviewer #1: No

Reviewer #2: No

**********

[NOTE: If reviewer comments were submitted as an attachment file, they will be attached to this email and accessible via the submission site. Please log into your account, locate the manuscript record, and check for the action link "View Attachments". If this link does not appear, there are no attachment files.]

To ensure your figures meet our technical requirements, please review our figure guidelines: https://journals.plos.org/plosone/s/figures

You may also use PLOS’s free figure tool, NAAS, to help you prepare publication quality figures: https://journals.plos.org/plosone/s/figures#loc-tools-for-figure-preparation.

NAAS will assess whether your figures meet our technical requirements by comparing each figure against our figure specifications.

Attachment Submitted filename: Review for PLOS ONE Paper.docx

Attachment Submitted filename: review_plos_one.pdf

# Author comment: Author response to Decision Letter 2

**DOI:** 10.1371/journal.pone.0359983.r004

17 Jul 2026

Review for PLOS ONE (PONE-D-26-17282R1)

Deep Learning-Based Predictions and Analysis of Norovirus Dynamics using a Fractional-order Mathematical Model

This manuscript proposes a fractional-order SVEIR model for norovirus transmission using the Caputo-Fabrizio derivative. The authors present fixed-point existence/uniqueness proofs, numerical simulations using a three-step Adams-Bashforth scheme, and a deep neural network (DNN) approximation with a novel activation function (NGNDG-AF). The manuscript addresses an interesting application of fractional calculus to norovirus modeling, and the existence/uniqueness analysis is a positive contribution. However, the current version contains technical errors. These issues must be addressed before the publication.

I recommend Minor Revision and encourage the authors to carefully address each of the points below.

1. The manuscript contains numerous grammatical errors and awkward phrasings make it correct

Author Response: Checked carefully and updated the manuscript file.

2. The abstract is overly long and repetitive. Many sentences could be condensed.

Author Response: We have removed the repetition and updated the manuscript file.

3. The fractional-order model is presented in integer-order form (Equation 1), but the analysis sand numerical methods assume a Caputo-Fabrizio formulation. The authors never explicitly write the fractional version of the system.

Author Response: We have clarified the implementation of the Caputo–Fabrizio fractional derivative in Equation (1). Specifically, the ordinary derivative has been replaced with the Caputo–Fabrizio fractional derivative, and the corresponding explanation has been added to the revised manuscript.

4. In Section 4 (Main Results), the integral representation uses a parameter `η` that is never clearly defined in relation to the fractional order `ζ`. This creates confusion throughout the existence/uniqueness proofs.

Author Response: We agree that the use of η and ζ created inconsistency. We have carefully checked the notation throughout the manuscript and revised it to ensure consistency.

5. The model includes disease-induced mortality `d = 0.33`, which is extremely high (33% of infected individuals die). Norovirus mortality is typically well below 1% in developed countries. This parameter likely represents something else or is grossly miscalibrated.

Author Response: The reported value d=0.33 is a typographical error in the parameter table. The correct value is d=0.02, as given in Ref. [23]. This error has been corrected in the revised manuscript.

6. Figure captions are missing thoroughly.

Author Response: Figures captions are thoroughly checked, highlighted and updated in the revised manuscript file.v

Review Report for PLOS ONE (PONE-D-26-17282R1)

Overall Decision: Major Revision Required

The manuscript attempts to combine fractional calculus (Caputo-Fabrizio), a compartmental model (SVEIR), numerical simulations (Adams-Bashforth), and Deep Neural Networks (DNN). While the topic is timely, the current execution suffers from lack of biological justification, mathematical inconsistencies, poor presentation of DNN results, and missing critical information (units, parameter sources, compartment diagram). The claimed "memory effect" of fractional order is not mechanistically linked to norovirus biology.

1) In introduction, the Motivation for fractional-order is weak and generic. The authors state fractional calculus captures "memory and nonlocal processes" but never explain what specific memory in norovirus transmission justifies a fractional derivative.

Author Response: Introduction has been updated with proper citations and highlighted in the revised manuscript file.

2) Norovirus infection lasts approx. 2 to 3 days in healthy individuals. What biological mechanism requires a memory kernel spanning weeks or months?

Author Response: This is the insightful observation from reviewer’s. We agree that the norovirus infection lasts approximately 2 to 3 days in healthy individuals. Basically, norovirous transmission dynamics involve memory related effects due to environmental viral persistence, asymptomatic transmission and influence of previous infections on future spread. Fractional order models capture these historical dependencies and and provide a more realistic description of novovirous transmission. Reference [23]

3) The statement "classical integer-order models characterize memory only to a limited extent" is vague. No example or citation is given where integer-order SEIR-type models failed for norovirus.

Author Response: We agree that the previous statement was too general. We have revised the manuscript to clarify that classical integer-order SEIR-type models are useful for describing norovirus transmission but assume that the current state depends only on instantaneous conditions and may not explicitly incorporate history-dependent effects such as environmental persistence, delayed transmission, and temporary immunity. Appropriate references have been added to support this clarification. Reference [24]

4) Caputo Fabrizio (CF) derivative uses an exponential kernel (no singularity), while Caputo has a power-law kernel. For disease with short infectious period, the biological rationale for CF over standard Caputo is not provided. The authors mention "Atangana–Baleanu fractional derivative" but then use CF. This is confusing and not justified.

Author Response: We know that Caputo, Caputo-Fabrizio and Atangana- Baleanu are different operators are distinct fractional operators characterized by different kernel functions and properties. The confusing statement has been removed from revised manuscript file.

5) The abstract and introduction repeat the same sentences almost verbatim (e.g., "extremely contagious and has a significant impact on the gastrointestinal tract").

Author Response: Removed the repeated sentences and updated in the manuscript file

6) The statement "Deep Neural Networks provide a flexible framework without discretization" is true in principle, but later the DNN is trained on data from Adams-Bashforth. That defeats the purpose — it’s not a truly independent solver.

Author Response: The proposed DNN is trained directly using the differential equations along with conditions. No discretized numerical solution is used during the training process. The Adams-Bashforth method is employed solely to validate the accuracy of the proposed DNN by comparing the resulting numerical solutions.

7) No mention of prior fractional models for norovirus (if any) to establish novelty.

Author Response: The present work is primarily motivated by the studies reported in [19, 22, 23], which emphasize the importance of fractional-order norovirus models and reveal the need for accurate and efficient computational approaches. Moreover, we develop a Deep Neural Network (DNN)-based framework to obtain reliable numerical solutions for the proposed model.

8) In Model formulation, schematic diagram for the proposed model is missing.

Author Response: The schematic diagram added in the revised file

9) In table, Parameter values are given without sources or units.

Author Response: Updated with unit and source

10) Vaccine efficacy is very high. Is that realistic for norovirus vaccines still in development? Citation needed.

Author Response: Yes, norovirus vaccines still in development. The vaccine efficacy parameter used in this study represents a hypothetical value adopted for mathematical modeling and development analysis. It is intended to investigate the potential impact of a highly effective vaccine on disease transmission rather than to reflect the efficacy of an approved vaccine. This assumption is consistnent with modeling studies that evaluate prospective vaccination strategies and is supported by the current literature on norovirus vaccine development.

We updated the introduction part with the following latest references related to vaccines development [10,11]. [10] Tan, Ming. "The Impact of Norovirus on Children and Adolescents: Implications for Ongoing Vaccine Development." Current Pediatrics Reports 13.1 (2025): 21. [11] Lindesmith, Lisa C., et al. "Norovirus GII. 4 serological repertoire in older adults after GII. 4 ancestral variant vaccination: a post-hoc analysis of a randomised, double-blind, phase 2 trial." The Lancet Microbe 7.6 (2026).

11) What is the use of theoretical theorems in this section? Theorems on existence, uniqueness, and Ulam-Hyers stability (Section 3, page 76 onward) are mathematically standard for fractional systems but are not connected to norovirus biology. They do not inform parameter estimation, control strategies, or predictions.

Author Response: we agree that the existence, uniqueness, and Ulam-Hyers stability theorems are standard mathematical results for fractional differential systems. However, their inclusion is essential to establish the mathematical well posedness of the proposed norovirus transmission model before performing numerical simulation. Specifically, the existence and uniqueness results guarantee that the model admits a well defined and physically meaningful solution, while the Ulam-Hyers stability analysis demonstrates that the solution remains stable under small perturbations in the initial condions or model parameters. These theoretical properties ensure that the predicted disease dynamics are mathematically reliable and that the subsequent numerical and DNN based results are based on a strong mathematical foundation.

12) In section 4 and 5, The DNN is NOT solving the fractional model directly. Is that correct? The authors write: "The datasets, obtained from the piecewise Adams-Bashforth numerical methods, were used to train the DNN" (page 90). This means the DNN is merely fitting the numerical solution, not approximating the fractional differential equation independently.

Author Response: The DNN solves the fractional model directly, as stated by the authors on the same page (90) ―the network learn purely from the ODEs structure and initial conditions‖. Therefore, the sentence highlighted by the reviewer, "The datasets, obtained from the piecewise Adams-Bashforth numerical methods, were used to train the DNN", is indeed contradictory. In response to the reviewer comment, we have removed this sentence from the revised manuscript to eliminate the inconsistency.

13) Equation (67) defines a residual loss, but the authors then admit they used Adams-Bashforth data. This seems a contradiction.

Author Response: This contradiction has already been addressed in response to the previous comment and has been corrected in the revised manuscript.

14) Also make sure all figures are explained well in the text.

Author Response: All Figures have been carefully reviewed, appropriately described and properly referenced in the text. The descriptions of results in terms of MSE and number of training epochs have been thoroughly checked and updated throughout the revised manuscript.

Attachment Submitted filename: Response to Reviewer 2.pdf

# Peer review: Decision Letter 2

**DOI:** 10.1371/journal.pone.0359983.r005

**Authors:** Asad Ullah

5 Aug 2026

Dear Dr. Ali,

Thank you for submitting your manuscript to PLOS ONE. After careful consideration, we feel that it has merit but does not fully meet PLOS ONE’s publication criteria as it currently stands. Therefore, we invite you to submit a revised version of the manuscript that addresses the points raised during the review process.

Please submit your revised manuscript by **Sep 19 2026 11:59PM** . If you will need more time than this to complete your revisions, please reply to this message or contact the journal office at plosone@plos.org. When you're ready to submit your revision, log on to https://www.editorialmanager.com/pone/ and select the 'Submissions Needing Revision' folder to locate your manuscript file.

- A letter that responds to each point raised by the academic editor and reviewer(s). You should upload this letter as a separate file labeled 'Response to Reviewers'.
- A marked-up copy of your manuscript that highlights changes made to the original version. You should upload this as a separate file labeled 'Revised Manuscript with Track Changes'.
- An unmarked version of your revised paper without tracked changes. You should upload this as a separate file labeled 'Manuscript'.

If you would like to make changes to your financial disclosure, please include your updated statement in your cover letter. Guidelines for resubmitting your figure files are available below the reviewer comments at the end of this letter.

If applicable, we recommend that you deposit your laboratory protocols in protocols.io to enhance the reproducibility of your results. Protocols.io assigns your protocol its own identifier (DOI) so that it can be cited independently in the future. For instructions see: https://journals.plos.org/plosone/s/submission-guidelines#loc-laboratory-protocols. Additionally, PLOS ONE offers an option for publishing peer-reviewed Lab Protocol articles, which describe protocols hosted on protocols.io. Read more information on sharing protocols at https://plos.org/protocols?utm_medium=editorial-email&utm_source=authorletters&utm_campaign=protocols.

As the corresponding author, your ORCID iD is verified in the submission system and will appear in the published article. PLOS supports the use of ORCID, and we encourage all coauthors to register for an ORCID iD and use it as well. Please encourage your coauthors to verify their ORCID iD within the submission system before final acceptance, as unverified ORCID iDs will not appear in the published article. *Only*  the individual author can complete the verification step; PLOS staff *cannot*  verify ORCID iDs on behalf of authors.

We look forward to receiving your revised manuscript.

Kind regards,

Asad Ullah, Postdoc

Academic Editor

PLOS One

Journal Requirements:

If the reviewer comments include a recommendation to cite specific previously published works, please review and evaluate these publications to determine whether they are relevant and should be cited. There is no requirement to cite these works unless the editor has indicated otherwise.

Please review your reference list to ensure that it is complete and correct. If you have cited papers that have been retracted, please include the rationale for doing so in the manuscript text, or remove these references and replace them with relevant current references. Any changes to the reference list should be mentioned in the rebuttal letter that accompanies your revised manuscript. If you need to cite a retracted article, indicate the article’s retracted status in the References list and also include a citation and full reference for the retraction notice.

**Additional Editor Comments:**

**Authors are required to follow the journal format in all aspects, including references, font size, line spacing and figures styles etc. The figures and tables must be in the standard resolution as per journal requirements.**

[Note: HTML markup is below. Please do not edit.]

Reviewers' comments:

Reviewer's Responses to Questions

**Comments to the Author**

Reviewer #1: All comments have been addressed

Reviewer #2: All comments have been addressed

**********

2. Is the manuscript technically sound, and do the data support the conclusions?

Reviewer #1: Yes

Reviewer #2: Yes

**********

3. Has the statistical analysis been performed appropriately and rigorously?

Reviewer #1: Yes

Reviewer #2: Yes

**********

4. Have the authors made all data underlying the findings in their manuscript fully available?

The PLOS Data policy

Reviewer #1: Yes

Reviewer #2: Yes

**********

5. Is the manuscript presented in an intelligible fashion and written in standard English?

Reviewer #1: Yes

Reviewer #2: Yes

**********

Reviewer #1: The manuscript is significantly improved in terms of presentation, clarification of the fractional-order formulation, parameter corrections, inclusion of a schematic model diagram, and better explanation of the deep neural network framework. The study presents a meaningful integration of fractional-order epidemiological modeling with deep learning techniques for norovirus dynamics.

However, a few minor issues still deserve attention before publication:

1.The manuscript should undergo one final round of English language proofreading, as a few grammatical errors, typographical mistakes, and awkward sentences remain.

2.Ensure that all mathematical symbols and notations are used consistently throughout the manuscript (e.g., fractional-order parameters and operator notation).

3.Some figure captions and figure references in the text could be made more descriptive to improve readability.

4.The conclusion may be strengthened by briefly highlighting the practical implications and limitations of the proposed model, together with possible future research directions.

Reviewer #2: The authors have fulfilled all of my comments. I see significant imorovement in the manuscript. I recommend the manuscript for publication.

**********

what does this mean?). If published, this will include your full peer review and any attached files.

If you choose “no”, your identity will remain anonymous but your review may still be made public.

**Do you want your identity to be public for this peer review?**  For information about this choice, including consent withdrawal, please see our Privacy Policy

Reviewer #1: No

Reviewer #2: No

**********

[NOTE: If reviewer comments were submitted as an attachment file, they will be attached to this email and accessible via the submission site. Please log into your account, locate the manuscript record, and check for the action link "View Attachments". If this link does not appear, there are no attachment files.]

To ensure your figures meet our technical requirements, please review our figure guidelines: https://journals.plos.org/plosone/s/figures

You may also use PLOS’s free figure tool, NAAS, to help you prepare publication quality figures: https://journals.plos.org/plosone/s/figures#loc-tools-for-figure-preparation.

NAAS will assess whether your figures meet our technical requirements by comparing each figure against our figure specifications.

# Author comment: Author response to Decision Letter 3

**DOI:** 10.1371/journal.pone.0359983.r006

20 Aug 2026

Response to Reviewers

1. The manuscript should undergo one final round of English language proofreading, as a few grammatical errors, typographical mistakes, and awkward sentences remain.

Author Response: The grammatical errors and awkward sentences have been carefully revised, and the manuscript has been thoroughly updated and proofread to improve its overall clarity, readability, and language quality.

2. Ensure that all mathematical symbols and notations are used consistently throughout the manuscript (e.g., fractional-order parameters and operator notation).

Author Response: All mathematical symbols and notations are used consistently throughout the manuscript.

3. Some figure captions and figure references in the text could be made more descriptive to improve readability.

Author Response: The figure captions and corresponding references in the text have been revised and made more descriptive to improve clarity, readability, and understanding of the presented results.

4. The conclusion may be strengthened by briefly highlighting the practical implications and limitations of the proposed model, together with possible future research directions.

Author Response: The Conclusion section has been revised and updated accordingly in the manuscript

# Peer review: Decision Letter 3

**DOI:** 10.1371/journal.pone.0359983.r007

**Authors:** Asad Ullah

6 Sep 2026

Dear Dr. Ali,

Thank you for submitting your manuscript to PLOS One. After careful consideration, we feel that it has merit but does not fully meet PLOS One’s publication criteria as it currently stands. Therefore, we invite you to submit a revised version of the manuscript that addresses the points raised during the review process.

==============================

**ACADEMIC EDITOR:**

==============================

Please submit your revised manuscript by Oct 21 2026 11:59PM. If you will need more time than this to complete your revisions, please reply to this message or contact the journal office at plosone@plos.org. When you're ready to submit your revision, log on to https://www.editorialmanager.com/pone/ and select the 'Submissions Needing Revision' folder to locate your manuscript file.

- A letter that responds to each point raised by the academic editor and reviewer(s). You should upload this letter as a separate file labeled 'Response to Reviewers'.
- A marked-up copy of your manuscript that highlights changes made to the original version. You should upload this as a separate file labeled 'Revised Manuscript with Track Changes'.
- An unmarked version of your revised paper without tracked changes. You should upload this as a separate file labeled 'Manuscript'.

If you would like to make changes to your financial disclosure, please include your updated statement in your cover letter. Guidelines for resubmitting your figure files are available below the reviewer comments at the end of this letter.

If applicable, we recommend that you deposit your laboratory protocols in protocols.io to enhance the reproducibility of your results. Protocols.io assigns your protocol its own identifier (DOI) so that it can be cited independently in the future. For instructions see: https://journals.plos.org/plosone/s/submission-guidelines#loc-laboratory-protocols. Additionally, PLOS One offers an option for publishing peer-reviewed Lab Protocol articles, which describe protocols hosted on protocols.io. Read more information on sharing protocols at https://plos.org/protocols?utm_medium=editorial-email&utm_source=authorletters&utm_campaign=protocols.

As the corresponding author, your ORCID iD is verified in the submission system and will appear in the published article. PLOS supports the use of ORCID, and we encourage all coauthors to register for an ORCID iD and use it as well. Please encourage your coauthors to verify their ORCID iD within the submission system before final acceptance, as unverified ORCID iDs will not appear in the published article. *Only*  the individual author can complete the verification step; PLOS staff *cannot*  verify ORCID iDs on behalf of authors.

We look forward to receiving your revised manuscript.

Kind regards,

Asad Ullah, Postdoc

Academic Editor

PLOS One

Journal Requirements:

If the reviewer comments include a recommendation to cite specific previously published works, please review and evaluate these publications to determine whether they are relevant and should be cited. There is no requirement to cite these works unless the editor has indicated otherwise.

Please review your reference list to ensure that it is complete and correct. If you have cited papers that have been retracted, please include the rationale for doing so in the manuscript text, or remove these references and replace them with relevant current references. Any changes to the reference list should be mentioned in the rebuttal letter that accompanies your revised manuscript. If you need to cite a retracted article, indicate the article’s retracted status in the References list and also include a citation and full reference for the retraction notice.

[Note: HTML markup is below. Please do not edit.]

Reviewers' comments:

Reviewer's Responses to Questions

**Comments to the Author**

Reviewer #1: All comments have been addressed

**********

2. Is the manuscript technically sound, and do the data support the conclusions?

Reviewer #1: Yes

**********

3. Has the statistical analysis been performed appropriately and rigorously?

Reviewer #1: Yes

**********

4. Have the authors made all data underlying the findings in their manuscript fully available?

The PLOS Data policy

Reviewer #1: Yes

**********

5. Is the manuscript presented in an intelligible fashion and written in standard English?

Reviewer #1: Yes

**********

Reviewer #1: The manuscript presents a mathematical model for studying the transmission dynamics of norovirus using a fractional-order approach. The use of the Caputo-Fabrizio derivative, Adams-Bashforth numerical scheme, and deep neural network provides an interesting and useful framework for the analysis. The mathematical analysis and numerical results are presented clearly, and the DNN results support the numerical findings.

The authors need to addressed the following comments

1 language improvement

2 consistency of mathematical notation

3 improvement of figure captions

4 strengthening of the conclusion.

Overall, the manuscript is well organized and the results are relevant to mathematical epidemiology. I have no major concerns. I recommend acceptance of the manuscript after minor editorial corrections.

**********

what does this mean?). If published, this will include your full peer review and any attached files.

If you choose “no”, your identity will remain anonymous but your review may still be made public.

**Do you want your identity to be public for this peer review?**  For information about this choice, including consent withdrawal, please see our Privacy Policy

Reviewer #1: No

**********

[NOTE: If reviewer comments were submitted as an attachment file, they will be attached to this email and accessible via the submission site. Please log into your account, locate the manuscript record, and check for the action link "View Attachments". If this link does not appear, there are no attachment files.]

To ensure your figures meet our technical requirements, please review our figure guidelines: https://journals.plos.org/plosone/s/figures

You may also use PLOS’s free figure tool, NAAS, to help you prepare publication quality figures: https://journals.plos.org/plosone/s/figures#loc-tools-for-figure-preparation.

NAAS will assess whether your figures meet our technical requirements by comparing each figure against our figure specifications.

# Author comment: Author response to Decision Letter 4

**DOI:** 10.1371/journal.pone.0359983.r008

19 Sep 2026

Response to Reviewers

The authors need to addressed the following comments

1 language improvement

Authors Response: We have carefully revised the manuscript to improve the overall language, grammar, sentence structure, and readability.

2 consistency of mathematical notation

Authors Response: All relevant mathematical symbols, variables, parameters, and equations have been checked and standardized accordingly.

3 Improvement of figure captions

Authors Response: We have revised and improved the captions of all figures to make them more descriptive, clear, and self-explanatory.

4 Strengthening of the conclusion.

We have strengthened the conclusion section by providing a clearer summary of the main findings, highlighting the significance of the proposed approach, and discussing the potential implications and future directions of the study.

Attachment Submitted filename: Response_to_Reviewers_auresp_4.pdf

# Peer review: Decision Letter 4

**DOI:** 10.1371/journal.pone.0359983.r009

**Authors:** Asad Ullah

21 Sep 2026

Deep Learning-Based Predictions and Analysis of Norovirus Dynamics using a Fractional-order Mathematical Model

PONE-D-26-17282R4

Dear Dr. Rehman,

We’re pleased to inform you that your manuscript has been judged scientifically suitable for publication and will be formally accepted for publication once it meets all outstanding technical requirements.

Within one week, you’ll receive an e-mail detailing the required amendments. When these have been addressed, you’ll receive a formal acceptance letter and your manuscript will be scheduled for publication.

An invoice will be generated when your article is formally accepted. Please note, if your institution has a publishing partnership with PLOS and your article meets the relevant criteria, all or part of your publication costs will be covered. Please make sure your user information is up-to-date by logging into Editorial Manager at Editorial Manager® and clicking the ‘Update My Information' link at the top of the page. For questions related to billing, please contact billing support.

If your institution or institutions have a press office, please notify them about your upcoming paper to help maximize its impact. If they’ll be preparing press materials, please inform our press team as soon as possible -- no later than 48 hours after receiving the formal acceptance. Your manuscript will remain under strict press embargo until 2 pm Eastern Time on the date of publication. For more information, please contact onepress@plos.org.

Kind regards,

Asad Ullah, Postdoc

Academic Editor

PLOS One

Additional Editor Comments (optional):

Reviewers' comments:

Reviewer's Responses to Questions

**Comments to the Author**

Reviewer #1: All comments have been addressed

**********

2. Is the manuscript technically sound, and do the data support the conclusions?

Reviewer #1: Yes

**********

3. Has the statistical analysis been performed appropriately and rigorously?

Reviewer #1: Yes

**********

4. Have the authors made all data underlying the findings in their manuscript fully available?

The PLOS Data policy

Reviewer #1: Yes

**********

5. Is the manuscript presented in an intelligible fashion and written in standard English?

Reviewer #1: Yes

**********

Reviewer #1: All the commenta are answered clearly and the response is satisfactory. Therefore it is recommend to proceed it for further process of publication.

**********

what does this mean?). If published, this will include your full peer review and any attached files.

If you choose “no”, your identity will remain anonymous but your review may still be made public.

**Do you want your identity to be public for this peer review?**  For information about this choice, including consent withdrawal, please see our Privacy Policy

Reviewer #1: No

**********

# Editor report: Acceptance letter

**DOI:** 10.1371/journal.pone.0359983.r010

**Authors:** Asad Ullah

PONE-D-26-17282R4

PLOS One

Dear Dr. Ali,

I'm pleased to inform you that your manuscript has been deemed suitable for publication in PLOS One. Congratulations! Your manuscript is now being handed over to our production team.

At this stage, our production department will prepare your paper for publication. This includes ensuring the following:

* All references, tables, and figures are properly cited

* All relevant supporting information is included in the manuscript submission,

* There are no issues that prevent the paper from being properly typeset

You will receive further instructions from the production team, including instructions on how to review your proof when it is ready. Please keep in mind that we are working through a large volume of accepted articles, so please give us a few days to review your paper and let you know the next and final steps.

Lastly, if your institution or institutions have a press office, please let them know about your upcoming paper now to help maximize its impact. If they'll be preparing press materials, please inform our press team within the next 48 hours. Your manuscript will remain under strict press embargo until 2 pm Eastern Time on the date of publication. For more information, please contact onepress@plos.org.

You will receive an invoice from PLOS for your publication fee after your manuscript has reached the completed accept phase. If you receive an email requesting payment before acceptance or for any other service, this may be a phishing scheme. Learn how to identify phishing emails and protect your accounts at https://explore.plos.org/phishing.

If we can help with anything else, please email us at customercare@plos.org.

Thank you for submitting your work to PLOS One and supporting open access.

Kind regards,

PLOS One Editorial Office Staff

on behalf of

Dr. Asad Ullah

Academic Editor

PLOS One
